# Integrative genomic analyses reveal candidate genes for the *Ur‐3* and *Ur‐7* rust resistance loci in common bean

**DOI:** 10.1002/tpg2.70253

**Published:** 2026-05-23

**Authors:** Alvaro Soler‐Garzón, Giseli Valentini, Marcial Pastor‐Corrales, Ruifeng He, Phillip N. Miklas

**Affiliations:** ^1^ Irrigated Agriculture Research and Extension Center Washington State University Prosser Washington USA; ^2^ Department of Plant Sciences and Plant Pathology Montana State University Bozeman Montana USA; ^3^ Soybean Genomics and Improvement Laboratory, United States Department of Agriculture, Agricultural Research Service Beltsville Maryland USA; ^4^ Grain Legume Genetics and Physiology Research Unit, United States Department of Agriculture, Agricultural Research Service Prosser Washington USA

## Abstract

Bean rust, caused by *Uromyces appendiculatus*, severely limits the productivity of common bean (*Phaseolus vulgaris* L.), an essential source of protein and micronutrients in human diets worldwide. Durable disease management relies primarily on host resistance; however, the molecular architecture of several key rust resistance loci remains unresolved. We sought to characterize the genomic structure of the *Ur‐3* and *Ur‐7* loci and develop associated molecular markers. An integrative genomic framework combining high‐resolution long‐read genome assemblies, recombinant inbred line populations, and single‐nucleotide polymorphism–based and *k*‐mer‐based genome‐wide association studies was used to fine‐map both loci. Extensive structural variation was revealed for the *Ur‐3* locus at the distal end of chromosome Pv11, whereas *Ur‐7* localized to a low‐recombination pericentromeric region of the same chromosome. A coiled‐coil nucleotide‐binding leucine‐rich repeat gene embedded within an NB‐ARC‐rich cluster was identified as the most likely candidate underlying *Ur‐3* (Pv11: 50,205,868–50,209,452 bases; USDA Rattler reference). In contrast, the refined *Ur‐7* interval contained a toll/interleukin‐1 receptor NLR gene as the leading candidate (Pv11: 36,604,755–36,611,252; UI 111 reference). Gene‐based markers developed from these candidates showed complete correspondence with rust phenotypes. Marker‐assisted selection for *Ur‐3* and *Ur‐7* will contribute to the development of durable rust resistance against the highly variable bean rust pathogen *U. appendiculatus*.

## Plain Language Summary

Common beans are an important source of protein, fiber, and micronutrients. Production in certain environments is limited by bean rust disease. There are genes in common bean that provide resistance to bean rust. Using integrative genomics, we identified candidate genes underlying two major rust resistance genes *Ur‐3* and *Ur‐7*. Candidate‐gene linked markers were developed to facilitate selection of these two rust resistance genes in breeding programs aimed to limit production losses from bean rust disease.

**Abbreviations**
CNL: coiled‐coil nucleotide‐binding site leucine‐rich repeat
GWAS: genome‐wide association study
MAS: marker‐assisted selection
RIL: recombinant inbred line
SNP: single‐nucleotide polymorphism
TNL: toll/interleukin‐1 receptor nucleotide‐binding site leucine‐rich repeat

## 1 INTRODUCTION

The fungus *Uromyces appendiculatus* (Pers.:Pers) Unger is an autoecious, macrocyclic pathogen that causes bean rust, one of the most important diseases limiting common bean (*Phaseolus vulgaris* L.) production worldwide (Liebenberg & Pretorius, 2010). Rust symptoms on common bean plants appear as circular reddish‐brown pustules filled with spores of the rust pathogen. Although epidemics occur sporadically, bean rust can become highly destructive under prolonged periods of warm, temperate, and humid conditions. Under favorable conditions, numerous pustules develop on leaves and pods, resulting in substantial reductions in seed yield and quality (Schwartz & Pastor‐Corrales, 1989). Common bean plays a critical role in global food security as a primary source of plant‐based protein and micronutrients for millions of people worldwide (Cichy et al., 2009; Nadeem et al., 2021; Viscarra‐Torrico et al., 2021).

Bean rust management integrates cultural practices, resistant cultivars, and, when necessary, fungicide control measures (Liebenberg & Pretorius, 2010). In common bean, rust resistance is primarily controlled by major dominant resistance genes. These genes, designated with the *Ur* symbol (Kelly et al., 1996), include *Ur‐3*, *Ur‐5*, *Ur‐7*, *Ur‐11*, and *Ur‐14* from the Middle American gene pool (Hurtado‐Gonzales et al., 2017; Miklas et al., 2000, 2002; Park et al., 2003; Stavely, 1984, 1990; Valentini, Hurtado‐Gonzales, et al., 2025a), and *Ur‐4*, *Ur‐6*, *Ur‐9*, *Ur‐12*, and *Ur‐13* derived from the Andean gene pool (Grafton et al., 1985; Jung et al., 1998; Liebenberg & Pretorius, 2004; Luannfinke et al., 1986). Recently, a novel rust resistance gene located on chromosome Pv04 was discovered in the Andean landrace G19833 (Valentini et al., 2025c). Among these loci, *Ur‐3*, *Ur‐4*, *Ur‐5*, *Ur‐6*, *Ur‐7*, and *Ur‐11* have been widely deployed in breeding programs aimed at developing rust‐resistant cultivars (Pastor‐Corrales, 2005; Valentini et al., 2025b).

Recent advances in long‐read sequencing technologies, including Oxford Nanopore and PacBio HiFi, have transformed genomic analysis in *P. vulgaris* by enabling chromosome‐scale assemblies that resolve complex and repetitive regions (Y. Wang et al., 2025; Alvarez‐Diaz et al., 2026). In parallel, *k*‐mer‐based approaches have emerged as powerful tools for identifying trait‐associated sequence variation, particularly for loci affected by presence–absence variation or structural complexity (Wiersma et al., 2024).

In this study, we dissect the genomic architecture of the *Ur‐3* and *Ur‐7* rust resistance loci in common bean using an integrative genomic framework. The *Ur‐3* locus, historically mapped to the distal end of chromosome Pv11, is difficult to resolve due to extensive underlying structural variation. The *Ur‐7* locus, also situated on Pv11, remains insufficiently defined at the molecular and genetic level. To overcome these challenges, we integrated long‐read genome assemblies, single‐nucleotide polymorphism (SNP) and *k*‐mer‐based genome‐wide association study (GWAS), linkage mapping, and genome haplotype analysis to (i) resolve structural variation, (ii) identify high‐confidence candidate resistance genes, and (iii) develop gene‐based markers to improve marker‐assisted selection (MAS) for rust resistance in common bean.

## 2 MATERIALS AND METHODS

### 2.1 Plant material

The Durango Diversity Panel (DDP) consists of landraces, breeding lines, and cultivars, representing pinto, great northern, pink, and small red market classes developed by North American breeding programs from the late 1930s to around 2010. These materials ultimately derive from race Durango of the Middle American gene pool (Singh et al., 1991). The DDP was previously used for GWAS of host resistance to *Bean common mosaic virus* (BCMV) (Soler‐Garzón et al., 2021a, 2021b).

A subset of 130 DDP accessions was used for GWAS to identify genomic regions associated with *Ur‐3* and *Ur‐7* in this study. The DDP comprises 184 lines; however, 54 lines were excluded: 12 lines with *Ur‐11* identified by Valentini et al. (2025a) were removed because their combination with *Ur‐3* or *Ur‐4* masks the phenotypic effect of *Ur‐7*, 19 lines exhibited segregation for rust reaction, and rust reactions were not evaluated for 23 lines due to lack of available seed.

Additionally, two previously developed F_5:7_ recombinant inbred line (RIL) populations were used to map the *Ur‐7* resistance gene. The first population was derived from a cross between ‘Orion’ and USPT‐WM‐12 (O12; *n* = 158), and the second population from a cross between ‘Buster’ and ‘Roza’ (BR; *n* = 140). O12 and BR were originally used to map QTL for resistance to white mold (caused by *Sclerotinia sclerotiorum*) (Vasconcellos et al., 2017) and abiotic stress tolerance (Trapp et al., 2015), respectively. The GWAS identified the *Ur‐7* gene in Orion (DDP153, great northern) and both *Ur‐3* and *Ur‐7* genes in Buster (DDP163, pinto). Phenotypic analyses of the RIL populations revealed that USPT‐WM‐12 possessed the *Ur‐3* gene, whereas no rust resistance genes were detected in the pink bean Roza (DDP127).

### 2.2 Phenotypic evaluation of common bean plants for reaction to bean rust

All DDP lines, RILs, parents, and control cultivars were grown under greenhouse conditions at USDA‐ARS, Beltsville, MD, in 12.7‐cm diameter pots with two plants per pot. Primary leaves were inoculated when they were 40%–60% expanded, approximately 7 days after sowing. To ensure successful inoculation and verify race specificity, differential cultivars with known rust resistance genes were included as controls: Aurora (*Ur‐3*), Early Gallatin (*Ur‐4*), GN 1140 (*Ur‐7*), PI 181996 (*Ur‐11*), and UI 114 susceptible to all known races of *U. appendiculatus*.

Plants were inoculated with spores of *U. appendiculatus* races 31‐22 (old race designation 67), which infect plants with the *Ur‐3* but not *Ur‐7*, and 22–52 (old race designation 108), which induces a hypersensitive reaction (HR) in plants carrying *Ur‐3* and a resistant reaction identified by a small pustule reaction in plants with *Ur‐7*. The new race designations are based on reactions across a set of 12 host differentials using a modified binary system (Pastor‐Corrales et al., 2010). Inoculum was prepared by suspending frozen urediniospores in 25 mL of water containing 0.01% Tween 20 (Thermo Fisher Scientific) to a final concentration of 2 × 10^4^ urediniospores mL^−^^1^. The suspension was applied to the abaxial surface of primary leaves using a cotton swab. After inoculation, plants were maintained in a mist chamber (20 ± 1°C; >95% relative humidity) for 18 h in darkness, then transferred to the greenhouse for symptom development.

Rust symptoms were evaluated 10–12 days post inoculation using the 1–6 disease severity scale described by Stavely and Pastor‐Corrales (1989), where 1 = *no visible rust symptoms*, 2 = *small necrotic or chlorotic spots without sporulation (HR), whereby f2 is used to describe fainter spots and 2+ larger spots*, 3 = *tiny sporulating pustules less than 0.3 mm in diameter*, 4 = *sporulating pustules 0.3–0.5 mm in diameter*, 5 = *sporulating pustules 0.5–0.8 mm in diameter*, and 6 = *sporulating pustules larger than 0.8 mm in diameter*.

Plants with scores between 1 and 3 were classified as resistant, whereas those scoring from 4 to 6 were considered susceptible.

### 2.3 Genome‐wide SNP and *k*‐mer discovery within the DDP

The WGS data for the DDP were downloaded from the NCBI Sequence Read Archive (SRA) under BioProject PRJNA386820. For SNP discovery, sequencing reads were processed using the fastp tool (Chen et al., 2018) for quality control and trimming, then indexed, aligned, and sorted against the *P. vulgaris* Durango UI 111 *v*1.0 reference genome (https://phytozome‐next.jgi.doe.gov/info/PvulgarisUI111_v1_1) using Bowtie2 *v*2.3.4 (Langmead & Salzberg, 2012) and SAMtools (Li et al., 2009) (Figure S1A,B). Variant calling was performed with the next‐generation sequencing experience platform (NGSEP *v*4.0.2) pipeline (Lobaton et al., 2018; Tello et al., 2023). After filtering for a minor allele frequency greater than 0.05, missing data below 20%, and excluding repetitive regions, a total of 2,009,530 biallelic SNP variants were obtained across the 11 chromosomes of common bean. These variants were then used for imputation with the *ImputeVCF* command, and variant annotation was performed using the *VCFAnnotate* command, with both commands implemented in NGSEP.

Subsequently, SNP‐based GWAS was conducted using a mixed linear model (MLM) implemented in the Genome Association and Prediction Integrated Tool (GAPIT *v*3.5) (Lipka et al., 2012). To account for kinship and population structure, an identity‐by‐state kinship matrix was estimated using the Efficient Mixed Model Association algorithm within GAPIT. Additionally, three principal components derived from the same analysis were included as covariates to further control population structure. A Bonferroni correction was applied, retaining a genome‐wide significance threshold of *α* = 0.05. Visualization of marker‐trait associations and *Q–Q* plots were performed using CMplot *v*3.62 (Yin, 2016).

The *k*‐mer dataset was generated using KGASflow (Corut & Wallace, 2024), a modular Snakemake‐based workflow that integrates multiple preprocessing steps, including sequencing read quality assessment with FastQC (Andrews, 2010), adapter trimming with Cutadapt (Martin, 2011), and *k*‐mer counting with KMC (Kokot et al., 2017). Filtered 31‐bp *k*‐mers were subsequently used to construct the presence/absence and kinship matrices for *k*‐mer‐based genome‐wide association analysis, which implements the kmersGWAS approach described by Voichek and Weigel (2020). To evaluate the consistency of population structure estimates across SNP and *k*‐mer markers, a Mantel test was performed using the vegan package (Dixon, 2003) in R. The correlation was assessed using Pearson's product‐moment coefficient with 999 permutations between the *k*‐mer presence/absence and the SNP‐based kinship matrices.

In total, the dataset comprised 84,864,858,339 canonical *k*‐mers (Figure S2A–D). The analysis employed a MLM implemented within kGWASflow to account for population structure and relatedness. Significant *k*‐mers were aligned using BLASTN against the reference genomes G19833, UI 111, Labor Ovalle, and Pv5‐593 (available in Phytozome *v*14: https://phytozome‐next.jgi.doe.gov/); the USDA Rattler assembly generated in this study (described below); and the Aurora (*Ur‐3*), GN 1140 (*Ur‐7*), and ‘Mexico 235′ (*Ur‐3* ^+^) assemblies reported by McClean et al. (2022).

### 2.4 USDA Rattler de novo assembly

The pinto bean cultivar USDA Rattler (Miklas et al., 2023), known to possess the *Ur‐3* gene, was selected for genome sequencing. To represent the genetic profile of the cultivar, high‐molecular weight DNA was extracted from leaf tissue of a single plant from each of six USDA Rattler selections, using Genomic DNA Extraction Kit (HMW, Magnetic Beads) (BioDynami). DNA sequencing for every single selection was performed using Oxford Nanopore Technologies (ONT) minION on an R10.4.1 flow cell (FLO‐MIN114) with the SQK‐LSK114 (Oxford Nanopore Technologies Ltd) library preparation kit, and runs were conducted for 72 h.

Raw Pod5 files were base‐called using Dorado *v*0.5.1 (model: dna_r10.4.1_e8.2_400 bps_sup@v4.2.0) on the Kamiak High‐Performance Computing Cluster at Washington State University. Adapters were trimmed, and reads were converted to FASTQ format using Samtools *v*1.21 (Li et al., 2009). Remaining adapter sequences and chimeric fragments were removed with Porechop *v*0.2.4 (https://github.com/rrwick/Porechop). Filtered reads were further processed using Fastplong *v*0.4.1 (Chen et al., 2018; https://github.com/OpenGene/fastplong) to trim low‐quality regions (*Q* < 10) and retain reads longer than 3 kb. FASTQ files from the six Rattler selections were merged to increase genome coverage and sequencing depth. Read quality was assessed using NanoPlot *v*1.0.0 (De Coster et al., 2018) to verify read length distributions and quality scores before assembly.

De novo assembly was performed using Hifiasm *v*0.25.0‐r726 (Cheng et al., 2024) with the Nanopore R10 parameter setting. Base‐level error correction was conducted in three polishing rounds. The first two rounds used Racon *v*1.5.0 (parameters: ‐m 8 ‐x ‐6 ‐g ‐8 ‐w 500) (https://github.com/lbcb‐sci/racon), aligning ONT reads back to the draft assembly to refine consensus accuracy. A final polishing step was performed using Medaka *v*2.1.0 (model: dna_r10.4.1_e8.2_400 bps_hac@v4.1.0) (https://github.com/nanoporetech/medaka). Assembly metrics, including N50 and total assembly size, were calculated using QUAST *v*5.3.0 (https://github.com/ablab/quast).

The USDA Rattler draft assembly was ordered and scaffolded using RagTag *v*2.1.0 (Alonge et al., 2022) based on contig alignments to *P. vulgaris* reference genomes available in Phytozome *v*13. Additionally, the completeness of the USDA Rattler genome assembly was evaluated using BUSCO *v*5.6.1 (Benchmarking Universal Single‐Copy Orthologs) (Manni et al., 2021) with the fabales_odb10 database, which includes 5366 conserved orthologs specific to the Fabales order.

### 2.5 USDA Rattler repeat and gene annotation

A custom transposable element (TE) library was constructed using RepeatModeler2 *v*2.0.3 (https://github.com/Dfam‐consortium/RepeatModeler) to characterize the repetitive regions of *P. vulgaris*. The analysis was performed on a concatenated dataset comprising three genome assemblies (G19833, UI 111, and USDA Rattler), which were merged into a single FASTA file and used to build a repeat database with the BuildDatabase utility implemented in the Dfam TE Tools package (https://github.com/Dfam‐consortium/TETools). This custom library was further integrated with the curated TE libraries reported by Gao et al. (2014) and the msRepDB database (Taxon Id 3885) (Liao et al., 2022) to improve classification coverage and annotation accuracy. Redundant sequences were removed using CD‐HIT *v*4.8.1 (https://github.com/weizhongli/cdhit), and potential protein‐coding contaminants were filtered with ProtExcluder (https://github.com/NBISweden/ProtExcluder) using transcriptome‐derived proteins from the UI 111 and G19833 reference genomes (Phytozome *v*13). Finally, RepeatMasker *v*4.1.4 was employed with the refined library to identify and classify repetitive elements across the USDA Rattler assembly.

Publicly available *P. vulgaris* RNA‐Seq datasets representing diverse tissues and developmental stages (Table S1) were downloaded from the NCBI SRA. Raw reads were aligned to the USDA Rattler assembly using HISAT2 *v*2.2.1 (Kim et al., 2019) with parameters –max‐intronlen 5000 and –rna‐strandness RF to optimize intron length detection and strand‐specific alignment. The resulting BAM files were sorted and indexed using SAMtools *v*1.16 (Li et al., 2009).

RNA‐Seq alignments and protein sequences from the *P. vulgaris* reference genomes G19833 and UI 111 were used as evidence for gene structure prediction within the GAIUS–AUGUSTUS–BRAKER3 pipeline (https://github.com/Gaius‐Augustus/BRAKER) executed in a Docker environment to ensure computational reproducibility (Gabriel et al., 2023). Functional annotation of the predicted genes was performed using InterProScan *v*5.59‐91 (Jones et al., 2014), integrating databases (Pfam, SMART, PANTHER, and SUPERFAMILY) to identify conserved protein domains, gene families, and biological functions.

### 2.6 Synteny analysis of the *Ur‐3* region

BLASTN was used to assess synteny among the long‐read‐based *P. vulgaris* genomes, USDA Rattler, Aurora, and Mexico 235, including the Pv5‐593 reference genome. BLAST alignments were filtered to retain only genomic fragments sharing at least 90% sequence identity, and the resulting syntenic relationships were visualized using the R package genoPlotR *v*0.8.11 (Guy et al., 2010).

### 2.7 Recombinant line screening within the *Ur‐7* region in RIL populations

The O12 and BR RIL populations were previously genotyped as detailed by Vasconcellos et al. (2017) and Trapp et al. (2015) using the BARCBean6K BeadChip assay, which includes 5398 SNPs (Song et al., 2015). Briefly, DNA was extracted from 20 mg of young leaf tissue collected from each RIL, parents, and the checks using the DNeasy 96 Plant Kit (Qiagen). Samples were obtained from single plants grown under greenhouse conditions at the USDA‐ARS, Prosser, WA.

Genotyping was conducted on the Illumina platform following the Infinium HD Assay Ultra protocol (Illumina Inc.) at the USDA‐ARS Soybean Genetics and Improvement Laboratory, Beltsville, MD. Recombinant lines were subsequently identified within the *Ur‐7* genomic region defined by genome‐wide association analyses and used to refine the genomic intervals associated with rust resistance.

### 2.8 T_m_‐shift marker design and validation

Allele‐specific primers targeting *Ur‐3* and *Ur‐7* candidate genes were designed using Primer3 (Untergasser et al., 2012). For the *Ur‐7* SNP marker, Ur‐7_Pv11_GeneR, GC‐rich tails of 14 and 6 bp were added to the forward primers Fa and Fb (favorable allele), respectively, following the T_m_‐shift protocol described by J. Wang et al. (2005). The previously reported SS68 SNP marker linked to *Ur‐3* (Hurtado‐Gonzales et al., 2017) was also adapted to the T_m_‐shift primer design method. In contrast, the newly developed Ur‐3_Pv11_GeneR marker was validated as a dominant marker (Table 1).

**TABLE 1:** Primers used to generate markers for *Ur‐3* and *Ur‐7* genes conditioning resistance to the common bean rust pathogen.

Id marker		Sequences	Gene	Ta (°C)	Location UI 111	Location G19833 *v*2	Location Rattler *v*1
Ur‐3_Pv11_GeneR	F	AGCGGTGTAAGAAAGAAGAGGGC	*Ur‐3*	68	N/A	N/A	Pv11: 50,205,846–50,205,934
	R	GCTTTCCAATTTCAGCTTTGTCCTATAT					
ss68	Fa	gcgggcagggcggc‐GGTATAATATTAAACGACCTCA		52	Pv11: 54,816,996	Pv11: 50,670,372	Pv11: 50,179,835
	R	GGCATGTAATATATGTGGACTT					
	Fb	gcgggc‐GGTATAATATTAAACGACCTCT					
Ur‐7_Pv11_GeneR	Fa	gcgggcagggcggc‐CGCTATTGAAGGGTCAGAAG	*Ur‐7*	63	Pv11: 36,610,984	Pv11: 31,536,658	Pv11: 32,816,896
	R	TCTTTCACCCAATCAACGATCT					
	Fb	gcgggc‐CGCTATTGAAGGGTCAGAAA					

Abbreviations: N/A, not applicable; Ta, annealing temperature.

PCR amplifications were performed in a 15 µL reaction containing 20 ng of genomic DNA, 1x Taq buffer, 1.5 mM MgCl_2_, 0.2 mM dNTP mix (Promega), 0.15 µM of each primer, 1x EvaGreen dye (Biotium), and 0.1 µL Taq DNA polymerase (Promega). An initial denaturation at 94°C for 2 min, followed by 35 amplification cycles consisting of 94°C for 20 s, annealing at the marker‐specific annealing temperature (Ta) for 20 s, and extension at 72°C for 20 s. A final extension step was performed at 72°C for 5 min.

Finally, melting curve analysis for allele discrimination was performed using a LightCycler 480 Instrument II (Roche) with EvaGreen fluorescent dye (Biotium). Fluorescent detection by 1 min at 95°C and the melting curve step ramping from 65°C to 95°C in increments of 1°C every 20 s.

## 3 RESULTS

### 3.1 Rust phenotyping

A subset of 130 from 184 DDP lines was evaluated phenotypically for response to two *U. appendiculatus* races, 31‐22 (67) and 22–52 (108). Nineteen (14.6%) DDP lines exhibited resistance to both rust races, 72 (55.4%) lines showed susceptibility to both rust races, and 39 (30%) lines exhibited resistance to race 22–52 and susceptibility to 31‐22 (Table S2). For the controls, Aurora with *Ur‐3* gene exhibited susceptibility to race 31‐22 and resistance to 22–52, and GN 1140 with *Ur‐7* gene showed resistance to both rust races. Additionally, parentals of the RIL populations Orion (*Ur‐7*) and Buster (*Ur‐3* and *Ur‐ 7*) exhibited rust resistance to both races. USPT‐WM‐12 (*Ur‐3*), like Aurora, exhibited susceptibility to 31‐22 and resistance to 22–52, and Roza exhibited susceptibility to both races. Phenotyping using multiple rust races revealed clear resistant and susceptible reactions among evaluated genotypes, consistent with race‐specific resistance responses.

### 3.2 USDA Rattler assembly

The USDA Rattler genome assembly comprised 1276 contigs with a total length of 611.2 Mb, a sequencing coverage of 40x, and a GC content of 36.2%. The assembly exhibited a high level of contiguity, with an N50 of 35.7 Mb, N90 of 5.1 Mb, and a largest contig of 60.9 Mb. Following the removal of 1212 organellar contigs (37.7 Mb), the refined assembly spanned of 573.5 Mb distributed across 64 scaffolds.

To improve continuity, contigs were ordered and oriented using the Pv5‐593 reference genome (Roy et al., 2025), selected for its low contig number and Middle American genetic background. Of the total assembly, 11 chromosomes accounted for 569.9 Mb and contained 2400 gap bases, while 29 unplaced scaffolds remained, comprising 3.6 Mb (Figure S3A,B). The assembly has been deposited in NCBI under BioSample accession SAMN54680171.

Assembly completeness, evaluated using BUSCO with the fabales_odb10 dataset, indicated a 99.3% complete genome. Repetitive elements accounted for 311.04 Mb (54.23%) of the USDA Rattler genome, with long terminal repeat lineages being the most abundant, particularly the Gypsy (25.6%) and Copia (21.2%) families (Figure S3C). Gene annotation of the soft‐masked genome identified 29,390 gene models (including isoforms), all of which were assigned to the 11 chromosomes (Figure S3D).

### 3.3 *Ur‐3* gene mapping and candidate genes

To validate our approach for identifying the *Ur‐3* locus, a Mantel test confirmed a significant correlation between the *k*‐mer and SNP‐derived kinship matrices (*r* = 0.56, *p* = 0.001). This statistical concordance demonstrates that both marker types consistently reflect the underlying population structure, thereby justifying the use of marker‐specific kinship matrices to control for genetic background across both GWAS platforms.

For the SNP‐based GWAS, qualitative rust reactions of each DDP accession were converted into a binary quantitative scale. Accessions like Aurora that were susceptible to race 31‐22 (67) and resistant to race 22–52 (108) were coded as 1 (*n* = 39; indicating the presence of *Ur‐3*), while those susceptible to both races were coded as 0 (*n* = 72; lacking *Ur‐3*). Additionally, 18 lines that showed resistance to both races, likely due to the presence of *Ur‐7*, were reclassified based on their rust reaction scores. Among these, five lines with a score of 2 or 2^+^ for race 22–52 (108) were coded as 1, whereas 13 lines with a score of 3 or f2 were coded as 0.

**FIGURE 1:**
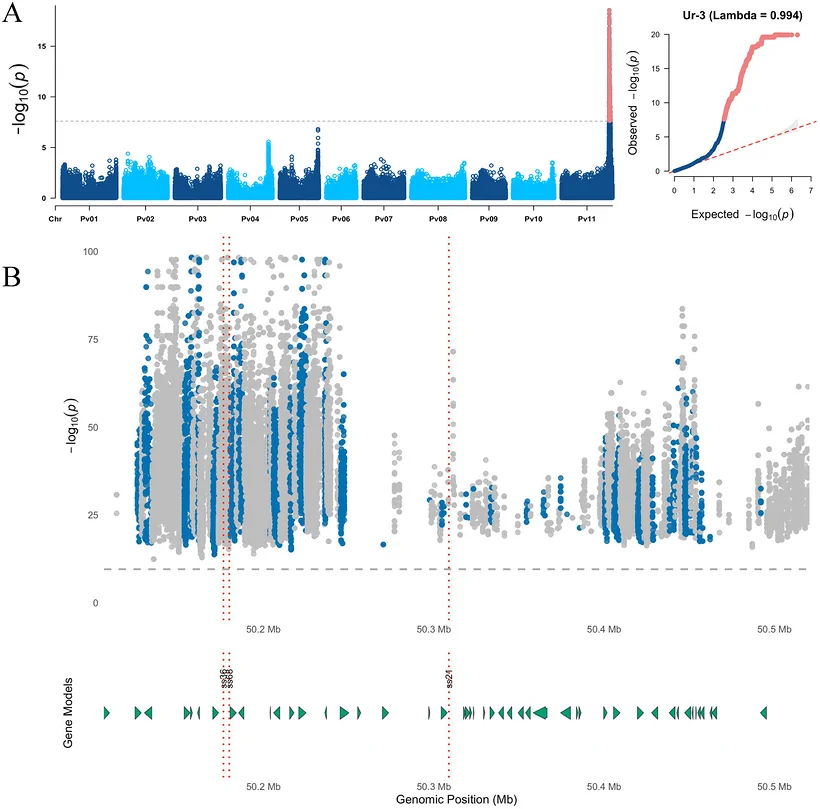
Detection of the *Ur‐3* resistance gene in common bean using single‐nucleotide polymorphism (SNP)‐based and *k*‐mer‐based genome‐wide association study (GWAS). (A) Manhattan plot showing significant SNPs genotyped in the Durango Diversity Panel and aligned to the UI 111 reference genome. Points exceeding the −log_10_ (*p*) Bonferroni correction threshold (gray dashed horizontal line) indicate significant associations with rust resistance on chromosome Pv11 and *Q*–*Q* plot of observed versus expected −log_10_ (*p*) values, with genomic inflation factor of 0.994. (B) Manhattan plot displaying a dense cluster of 27,349 significant *k*‐mers (blue circles in genes, and gray circles in intergenic regions) associated with the *Ur‐3* gene conferring rust resistance on Pv11 in the USDA Rattler reference genome. Red dashed vertical lines mark the flanking markers previously identified by Hurtado‐Gonzales et al. (2017) in an F_2_ population (ss36, ss68, and ss21, respectively).

A significant association interval was identified on chromosome Pv11 of the UI 111 reference genome, spanning 54,428,699–56,266,309 bp (approximately 1.8 Mb). This region exceeded the Bonferroni‐corrected significance threshold (*α* = 0.05; *p* ≤ 2.5 × 10^−^^8^) and contained a peak SNP located at 54,822,511 bp, which was significantly associated with resistance to the rust pathogen race 22–52 (108) (Figure 1A). The genomic inflation factor (*λ*) for GWAS was 0.994, confirming that population structure and relatedness were well‐controlled. This genomic interval overlapped with the *Ur‐3* region between 50,666,562 and 50,716,653 bp (approximately 0.5 Mb) in the Andean G19833 *v*2.1 reference genome reported by Hurtado‐Gonzales et al. (2017), using fine mapping in an F_2_ population.

Using the same binary phenotypic data associated with the *Ur‐3* gene in the DDP lines, the *k*‐mer‐based GWAS identified 99,966 *k*‐mers significantly associated with rust resistance (*α* = 0.05; *p* ≤ 6.06 × 10^−10^) (Figure S4A). Among these, 90,356 *k*‐mers (90%) were identified in the USDA Rattler assembly with 100% sequence identity and coverage. Similarly, 87,706 *k*‐mers (87.7%) were identical in the Aurora genome, while 48,379 (48.4%) *k*‐mers showed complete identity in the Mexico 235 genome. As expected, the reference genomes Labor Ovalle, Pv5‐593, GN 1140, G19833, and UI 111, which lack the *Ur‐3* gene, displayed lower alignment rates, ranging from 22% to 8% of identical *k*‐mers (Figure S4B). Additionally, a total of 46,487 *k*‐mers were shared among the USDA Rattler, Aurora, and Mexico 235 genomes (Figure S4C), of which 27,349 *k*‐mers were mapped to the USDA Rattler assembly and located between flanking markers reported in a previous study (Figure 1B).

**FIGURE 2:**
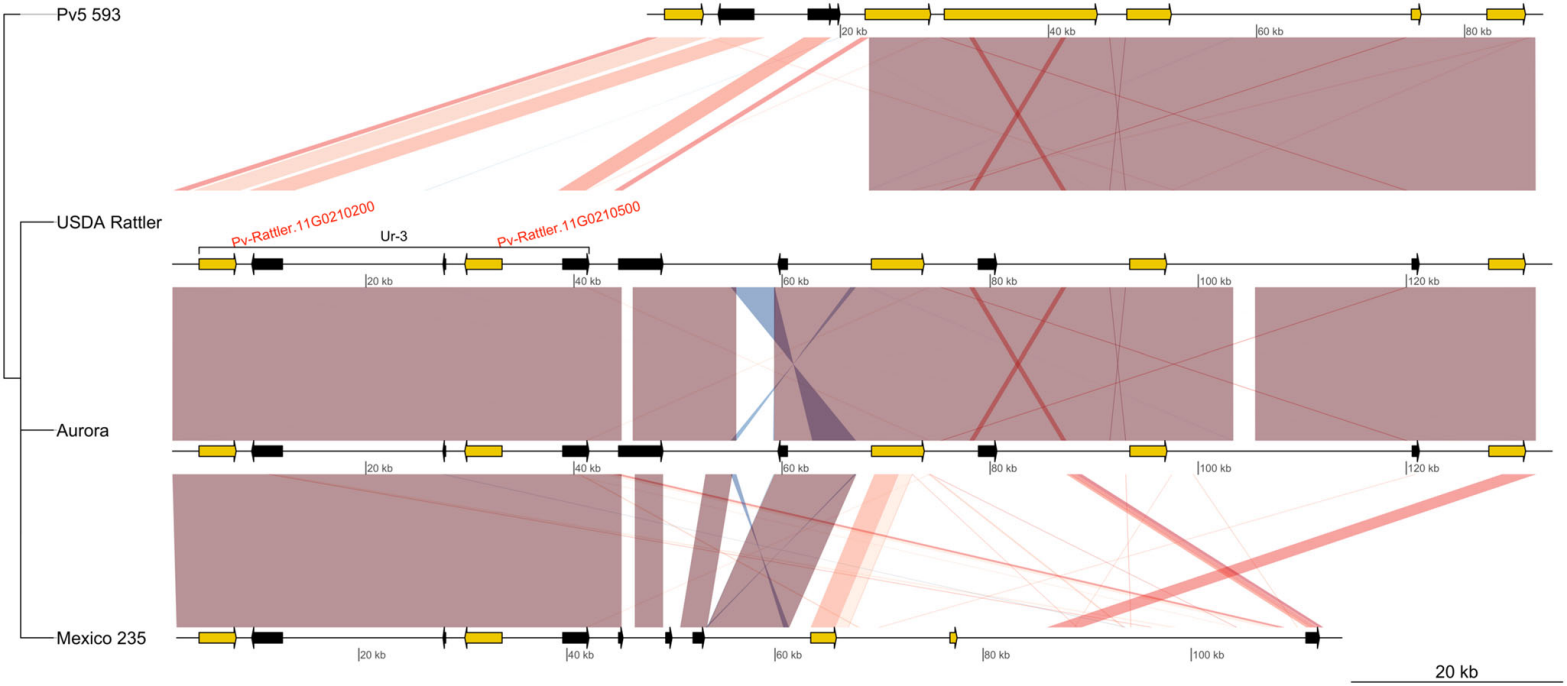
Comparative gene map of the *Ur‐3* region in Pv5‐593, USDA Rattler, Aurora, and Mexico 235. The tree on the left shows the relationship among the four genotypes. Shaded connections represent BLAST alignments between genomes with an identity >90%. Genes are shown as arrows indicating transcriptional direction. Yellow arrows represent NB‐ARC/leucine‐rich repeat (LRR) genes with disease‐resistant function.

Synteny analysis among the Pv5‐593 (*ur‐3*), USDA Rattler (*Ur‐3*), Aurora (*Ur‐3*), and Mexico 235 (*Ur‐3* ^+^) genomes revealed several genomic insertions within the *Ur‐3* region (Figure 2). The size of the *Ur‐3* locus was defined using the flanking SNP markers SS32 (Pv11: 54,367,946 bp; Pv5‐593 reference) and SS21 (Pv11: 54,454,705 bp; Pv5‐593 reference), as reported by Hurtado‐Gonzales et al. (2017). The estimated *Ur‐3* region was 86.8 kb in Pv5‐593, approximately 132.4 kb in USDA Rattler and Aurora (including a 6.8 kb gap), and 88.8 kb in Mexico 235 (with an 8.4 kb gap). The same 12 gene models were identified within the *Ur‐3* homologous region in USDA Rattler (Pv11: 50,176,368–50,308,766 bp) and Aurora, while only five of the 12 models were observed for the *Ur‐3* region in Mexico 235 (Table 2).

**TABLE 2:** Within the 12‐gene Ur‐3 interval, a candidate gene Pv‐Rattler.11G0210500 (bold type) for the *Ur‐3* gene in the USDA Rattler v1.0 reference genome, conditioning resistance to race 22–52 (108) of the bean rust pathogen, is conserved across the Aurora and Mexico 235 genomes, which also possess *Ur‐3*, and is absent in 5–593 genome, which lacks *Ur‐3*.

Gene model	Chr.	Position	*E*‐value	*Arabidopsis thaliana*	Signature description
Pv‐Rattler.11G0210200	Pv11	50,180,330–50,183,902	2e‐176	AT3G14470	CC‐NB‐ARC domain/LRR
Pv‐Rattler.11G0210300	Pv11	50,185,390–50,188,348	2e‐162	AT4G23180	CRK10/salt stress response/antifungal
Pv‐Rattler.11G0210400	Pv11	50,203,770–50,204,033	2e‐20	AT1G11410	S‐locus lectin protein kinase family protein/protein Kinases, catalytic domain
**Pv‐Rattler.11G0210500**	Pv11	50,205,868–50,209,452	1e‐155	AT3G14470	CC‐NB‐ARC domain/LRR
Pv‐Rattler.11G0210600	Pv11	50,215,282–50,217,812	7e‐167	AT4G23180	CRK10/salt stress response/antifungal
Pv‐Rattler.11G0210700	Pv11	50,220,652–50,224,928	6e‐08	AT3G14470	Rx N‐terminal domain
Pv‐Rattler.11G0210800	Pv11	50,235,968–50,236,882	–	–	CCHC‐type domain‐containing protein‐related
Pv‐Rattler.11G0210900	Pv11	50,244,946–50,250,018	9e‐152	AT3G14470	NB‐ARC domain/LRR
Pv‐Rattler.11G0211000	Pv11	50,255,218–50,256,999	–	–	LRR of kinetochore protein Cenp‐F/LEK1
Pv‐Rattler.11G0211100	Pv11	50,269,774–50,273,343	7e‐168	AT3G14470	NB‐ARC domain/LRR
Pv‐Rattler.11G0211200	Pv11	50,296,917–50,297,611	7e‐10	AT3G14470	Rx N‐terminal domain
Pv‐Rattler.11G0211300	Pv11	50,304,282–50,307,815	2e‐175	AT3G14470	NB‐ARC domain/LRR

Among the five gene models conserved across the *Ur‐3* region in USDA‐Rattler, Aurora, and Mexico 235 genomes, two encoded leucine‐rich repeat (LRR) proteins containing NB‐ARC and Rx N‐terminal domains (Pv‐Rattler.11G0210200 and Pv‐Rattler.11G0210500), two encoded cysteine‐rich receptor‐like kinase 10 (CRK10) proteins (Pv‐Rattler.11G0210300 and Pv‐Rattler.11G0210600), and one encoded a serine/threonine kinase with a BRI1‐associated kinase 1–like (STK_BAK1‐like) catalytic domain (Pv‐Rattler.11G0210400). Of the 46 most significant *k*‐mers identified by *k*‐mer‐based GWAS in the *Ur‐3* region (*α* = 0.05; *p* ≤ 1.62 × 10^−82^) supported by flanking markers, eight *k*‐mers were associated with these five conserved gene models across Rattler, Aurora, and Mexico 235. Seven of these *k*‐mers mapped to noncoding regions, and one *k*‐mer was located within the coding sequence of the fourth gene model (Pv‐Rattler.11G0210500), an NB‐ARC gene predicted to play a role in disease resistance. BLASTp identified Phvul.011G193100 as the top NB‐ARC paralog in Andean G19833 *v*2.1 reference genome, with 78% identity and an *E*‐value of 0.

### 3.4 *Ur‐7* gene mapping and candidate genes

**FIGURE 3:**
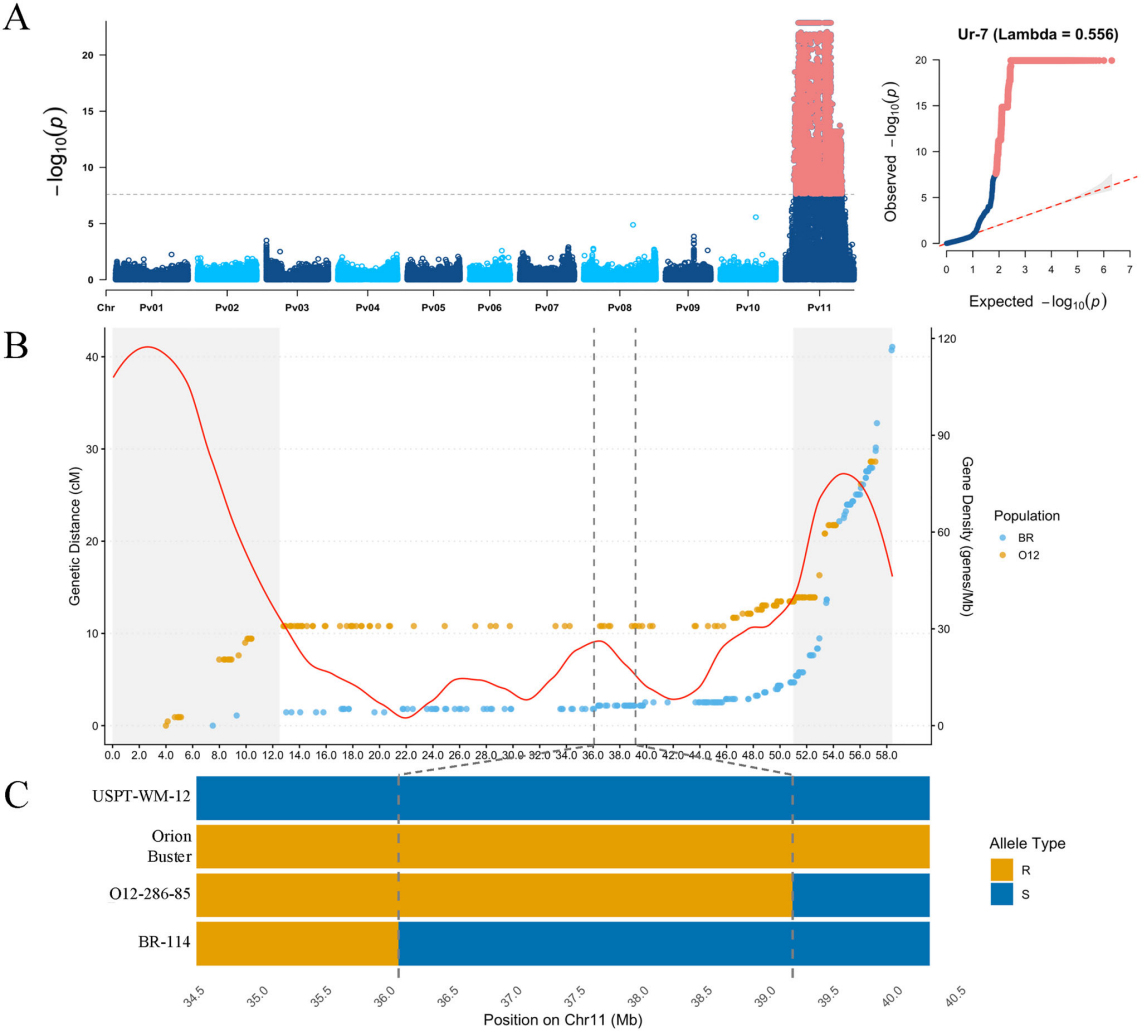
*Ur‐7* region detection and characterization in the Durango Diversity Panel (DDP) of common bean and recombinant inbred line (RIL) populations. (A) Manhattan plot of single‐nucleotide polymorphisms (SNPs) genetic markers associated with the *Ur‐7* gene, genotyped in the DDP and aligned to the UI 111 reference genome. SNPs above the −log_10_ (*p*) Bonferroni correction threshold (horizontal gray dashed line) indicate significant associations with rust resistance on chromosome Pv11, and *Q*–*Q* plot of observed versus expected −log_10_ (*p*) values, with genomic inflation factor of 0.556. (B) RILs population. Based on the comparison between physical distance (*X* axis) with genetic distance and average of genetic distance (blue and orange filled circles, left *Y* axis), and gene density (red line, right *Y* axis). Shaded euchromatic regions in gray and dashed vertical lines in gray indicate breakpoints in recombinant lines for *Ur‐7*. (C) Parental and recombinant haplotypes in the *Ur‐7* region (orange color = allele from *Ur‐7* parental donor and blue color = susceptible allele).

For the *Ur‐7* SNP‐based GWAS, 18 DDP lines exhibiting resistance to both rust races of *U. appendiculatus* were coded as 1 (*Ur‐7* presence), whereas 72 lines susceptible to both races and 39 lines showing susceptibility to race 31‐22 (67) and resistance to race 22–52 (108) were coded as 0 (lacking *Ur‐7*). A significant genomic interval (*α* = 0.05; *p* ≤ 2.5 × 10^−^^8^) was detected on chromosome Pv11, spanning 7,391,927–46,453,424 bp (39.1 Mb) in the UI 111 reference genome. The genomic inflation factor (*λ*) for this analysis was 0.556, indicating a highly conservative model that effectively controlled for population structure. Within this region, a narrower peak (*p* ≤ 1.36 × 10^−23^) between 10,975,133 and 38,843,386 bp (27.87 Mb) was observed (Figure 3A).

Additionally, *k*‐mer‐based GWAS identified 72,283 significant unique *k*‐mers (*α* = 0.05; *p* ≤ 6.06 × 10^−10^) in the GN 1140 genome known to possess the *Ur‐7* resistance gene (Park et al., 2003) (Figure S5A). Across other reference genomes lacking *Ur‐7*, significant *k*‐mers associated with *Ur‐7* accounted for 17%–23% of the total *k*‐mers detected (Figure S5B). Consistent with the SNP‐based GWAS results, these *k*‐mers in GN 1140 were distributed across 640 contigs, totaling 28.8 Mb in length (Figure S5C).

Based on analyses of the *Ur‐7* locus in O12 and BR RIL populations, low recombination rates were estimated at 10.1 and 11.5 Mb per cM, respectively, typical of pericentromeric regions. Similarly, reduced gene density for the *Ur‐7* region in the pericentromeric region (16.4 genes/Mb) was observed for chromosome Pv11 in the UI 111 reference genome, compared to higher density at the chromosomal ends (87.4 and 67.5 genes/Mb) (Figure 3B, upper).

Fine mapping using both RIL populations identified two recombinant lines, O12‐286‐85 (resistant) and BR‐114 (susceptible), which delimited the *Ur‐7* locus to a 3.05 Mb region spanning 36,070,200–39,176,174 bp (Figure 3B, bottom). Integrating SNP‐based GWAS and RIL fine‐mapping data further refined this interval to 36,070,200–38,843,386 bp (2.8 Mb). Within this region, 38 gene models were identified, including two gene models, PvUI111.11G144300 and PvUI111.11G144400, predicted to encode disease resistance‐related proteins.

**FIGURE 4:**
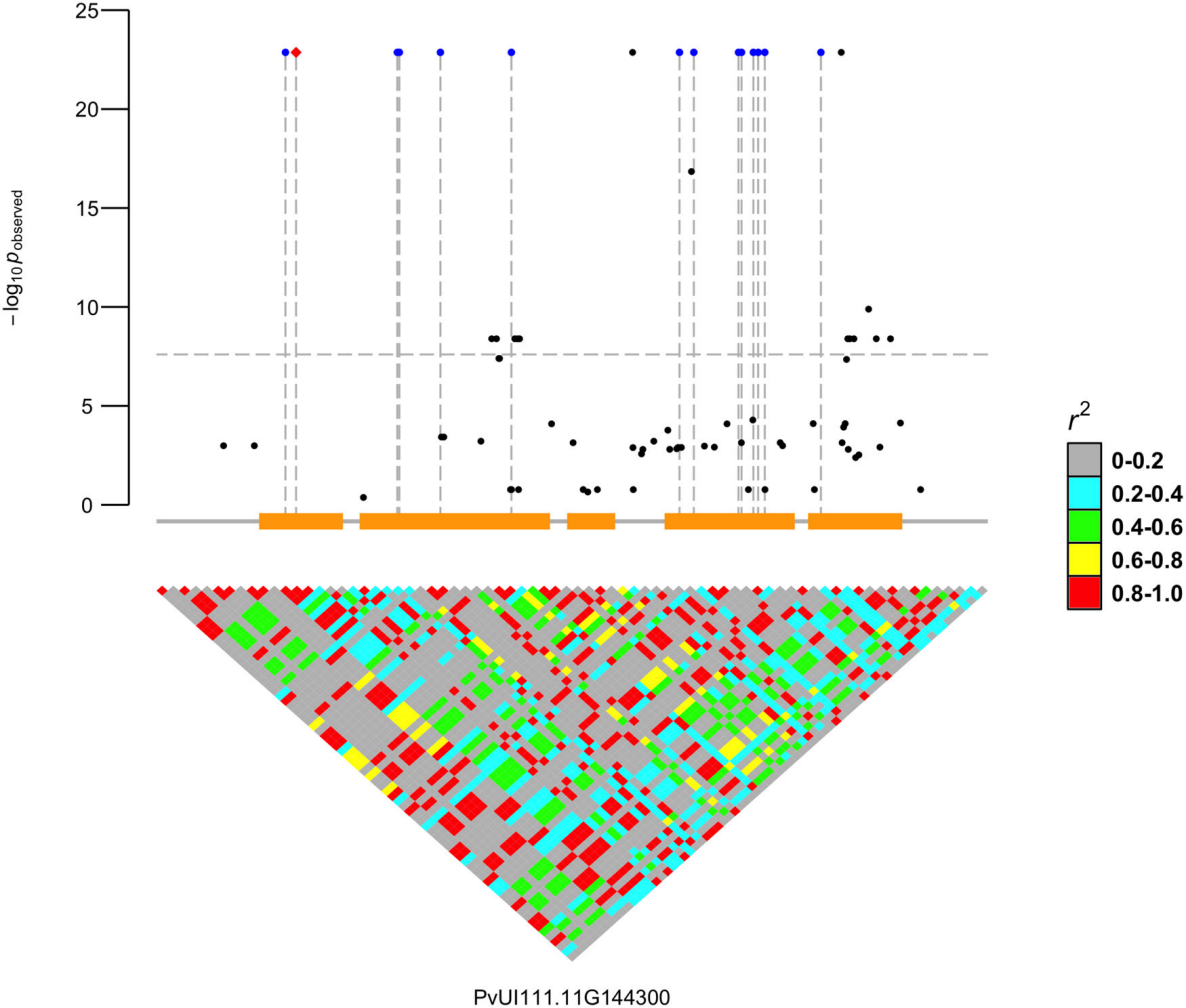
*Ur‐7* regional marker‐trait association and LD plot for the *PvUI111.11G144300* gene model. Single‐nucleotide polymorphisms (SNPs) are blue color‐coded, indicating the lead SNP based on SNP‐based genome‐wide association study (GWAS), with the vertical dashed line indicating the significance threshold (*α* = 0.05; *p* ≤ 2.5 × 10^−^^8^). Below, the gene structure shows exons (orange rectangles), introns (lines), and transcription direction. A triangular linkage disequilibrium (LD) heatmap highlights local LD patterns, enabling visualization of the relationship between significant SNPs and the *PvUI111.11G144300* gene.

A homolog of PvUI111.11G144300 was identified in the GN 1140 genome. This homolog contained 37 missense SNPs and two indel variants, and the SNP variants exhibited significant association with *Ur‐7* in DDP (Figure 4). In contrast, the neighboring gene PvUI111.11G144400 was not polymorphic. The PvUI111.11G144300 gene model encodes a protein composed of 1068 amino acids in UI 111 (*ur‐7*) and 1072 amino acids in GN1140 (*Ur‐7*). Importantly, the PvUI111.11G144300 gene–protein structure is homologous to the *Arabidopsis thaliana* gene AT5G17680 (*E*‐value = 1.59 × 10^−151^; identity = 35%) and Andean *P. vulgaris* G19833 *v*2.1 gene Phvul.011G140300 (*E*‐value = 0; identity = 77%), and contains canonical NB‐ARC, toll/interleukin‐1 receptor (TIR), LRR, and C‐JID domains characteristic of disease resistance proteins.

Beyond the primary toll/interleukin‐1 receptor nucleotide‐binding site leucine‐rich repeat (TNL) candidate PvUI111.11G144300, functional annotation of the remaining 36 gene models within the *Ur‐7* interval identified various functions related to stress response. These included roles in secondary metabolism, such as anthocyanidin 3‐O‐glucosyltransferases and glucose–methanol–choline oxidoreductases; cell signaling, involving late embryogenesis abundant proteins and cyclic nucleotide‐gated ion channels; and transcriptional regulation, including TCP10 transcription factors and pentatricopeptide repeat proteins.

### 3.5 *Ur‐3*‐ and *Ur‐7*‐linked marker assays

Two marker assays were developed for the candidate genes *Ur‐3* and *Ur‐7*, corresponding to Pv‐Rattler.11G0210500 and PvUI111.11G144300, respectively. The *Ur‐3* marker, named Ur3_Pv11_GeneR, was designed based on the USDA Rattler reference genome and is located at position Pv11: 50,205,846–50,205,934 within the Pv‐Rattler.11G0210500 candidate gene. The previous SS68 SNP marker for *Ur‐3* was mapped at Pv11: 50,179,835 bases in the USDA Rattler reference in an intergenic region upstream of Pv‐Rattler.11G0210200.

The *Ur‐7* marker, named Ur7_Pv11_GeneR, was designed using the PvUI111.11G144300 gene model from the UI 111 genome, which has a homologous gene in the *Ur‐7*‐resistant GN 1140 line. The Ur7_Pv11_GeneR marker targets a missense variant located in the first exon of the *Ur‐7* candidate gene at position Pv11: 36,610,984 bases in the UI 111 reference genome.

### 3.6 Survey of the markers in dry beans

A total of 177 dry bean accessions were surveyed using *Ur‐3* and *Ur‐7* gene‐based markers. The Ur3_Pv11_GeneR and Ur7_Pv11_GeneR markers achieved 100% correlation with observed rust phenotypes. Genotyping with the *Ur‐3* marker, Ur3_Pv11_GeneR, identified 56 lines (31.64%) carrying the favorable *Ur‐3* allele. Results from this new marker assay are an improvement on the previous SS68 allele assay which showed four susceptible lines (BFS 94, Labor Ovalle, PR‐0737‐1, and PR‐1146‐123) to possess *Ur‐3* resistance. These lines were confirmed to lack the *Ur‐3* gene based on susceptible reactions to bean rust race 22–52 (108) (Table S3).

The favorable *Ur‐7* allele, Ur7_Pv11_GeneR, was detected in 22 accessions, representing 12.43% of the panel. These included 19 DDP lines that were resistant to both races, as well as the cultivars CO46348, Norstar, and Olathe. Olathe is a differential line for *Ur‐6^+^*, exhibiting broader rust resistance than genotypes carrying *Ur‐*6 alone (Valentini et al., 2025c). Kelly et al. (1996) reported an additional rust resistance gene, *Urc*, in Olathe and proposed that *Urc* is equivalent to *Ur‐7*. Consistent with these findings, our results suggest that *Ur‐7* acts in combination with *Ur‐6* to confer the enhanced rust resistance observed in the Olathe cultivar.

## 4 DISCUSSION

Deploying host resistance genes remains the most effective and economically viable strategy for managing bean rust disease in common bean. The development of durable resistance is facilitated by the accurate identification and combination (pyramiding) of well‐characterized resistance loci into target breeding lines through MAS. High‐resolution mapping, sequence‐based allele discovery, and comparative approaches enable the refinement of the genetic architecture of major rust resistance genes and the development of robust markers to accelerate breeding. In this work, we integrated modern genomic approaches, including SNP‐ and *k*‐mer‐based GWAS, RIL genotyping, and multiple next‐generation sequencing, to pinpoint markers within strong candidate genes to improve MAS of the *Ur‐3* and *Ur‐7* rust resistance genes.

The significant correlation (*r* = 0.56) between SNP and *k*‐mer kinship matrices likely reflects the biological differences between point mutations and structural variations. By leveraging these complementary marker systems, we identified a narrowed interval for the *Ur‐3* locus, located, as in previous studies, at the distal end of chromosome Pv11. Further examination of this locus across previously sequenced genotypes revealed extensive structural variation. To further characterize this variation, we generated a chromosome‐level assembly of the USDA Rattler pinto bean, which possesses the *Ur‐3* resistance gene. The USDA Rattler assembly also provides a valuable genomic resource for future breeding and functional studies. The USDA Rattler genome complements the four existing *P. vulgaris* reference genomes available in Phytozome (https://phytozome‐next.jgi.doe.gov) and 13 other *P. vulgaris* genome assemblies currently accessible through NCBI (https://www.ncbi.nlm.nih.gov/datasets/genome/?taxon=3885) (Schmutz et al., 2014; Vlasova et al., 2016; Y. Wang et al., 2025; Perry et al., 2013; Jurado et al., 2025; Roy et al., 2025; Alvarez‐Diaz et al., 2026).

Comparative analysis of the structural variation for the *Ur‐3* interval across USDA Rattler and select genotypes with *Ur‐3* and without *ur‐3* resistance revealed a single candidate gene, the NBS‐ARC‐LRR gene Pv‐Rattler.11G0210500, within a cluster of five genes encoding disease resistance proteins that were conserved in genomes possessing *Ur‐3*. However, of the five genes, only Pv‐Rattler.11G0210500 was absent in rust‐susceptible genotypes. Previously, the SNP marker SS68 was reported to be linked to *Ur‐3*; however, SS68 is in an intergenic region (Hurtado‐Gonzales et al., 2017) within the five‐gene cluster identified in this study. Although SS68 was initially considered a reliable marker, false‐positive genotypes were identified in the present study. For example, Labor Ovalle, a black‐bean landrace from Guatemala, with a genome assembly in Phytozome (https://phytozome‐next.jgi.doe.gov/info/PvulgarisLaborOvalle_v1_1), carried the favorable SS68 SNP allele, but was found in our inoculations to be susceptible to 31‐1 (53) and 22–52 (108) races that *Ur‐3* is effective against. Conversely, the new candidate gene marker Ur3_Pv11_GeneR, which targeted an InDel within Pv‐Rattler.11G0210500, showed complete co‐segregation with the *Ur‐3* resistance phenotype across 56 bean genotypes. This marker functions as a dominant marker, amplifying only in resistant lines carrying *Ur‐3* because the candidate gene is absent in susceptible genotypes.

*Ur‐7* was consistently localized approximately 17.3 Mb distal to *Ur‐3* on Pv11, within a recombination‐suppressed interval. This localization is supported by a well‐calibrated model, as indicated by a genomic inflation factor (*λ*) of 0.994 for *Ur‐3* and 0.556 for *Ur‐7*. Additionally, GN 1140 was identified as carrying the *Ur‐7* haplotype, and *k*‐mer GWAS results indicated that it is the likely source of the resistance allele. *Ur‐7* was first mapped in an F_2_ population by Park et al. (2003), and a linked SCAR marker was later developed through bulked segregant analysis (Park et al., 2008). In the present study, this SCAR marker mapped approximately 2.3 Mb upstream of the refined *Ur‐7* interval on Pv11, placing it outside the candidate gene region. Together, these results improve understanding of the *Ur‐7* locus by pointing to a single TNL candidate gene and by providing a new SNP marker that can be readily used in breeding programs.

In the regions corresponding to the *Ur‐3* and *Ur‐7* loci, NB‐LRR‐type resistance genes were identified. Genotypes carrying either gene can express complete resistance or a HR to specific rust races. However, *Ur‐3* genotypes appear to trigger the HR across a broader range of rust races than *Ur‐7* genotypes (Valentini et al., 2025b). The primary structural difference between the two is that the *Ur‐7* candidate encodes a TNL protein, containing an N‐terminal TIR domain, while the *Ur‐3* candidate encodes a coiled‐coil NB‐LRR (CNL) protein with an N‐terminal coiled‐coil domain. TNL and CNL proteins typically function as intracellular immune receptors that detect pathogen effectors (or the alterations these effectors induce in host proteins), activating a localized hypersensitive cell‐death response to limit pathogen growth (Eitas & Dangl, 2010; Meyers et al., 1999; Van Ooijen et al., 2008). Consistent with this functional role, TNL genes have also been associated with resistance to Asian soybean rust, caused by *Phakopsora pachyrhizi*, in soybean (Bish et al., 2024). Similarly, CNL genes were associated with broad‐spectrum bean rust resistance in the Andean landrace G19833 (Valentiniet al., 2025c).

The identification of the *Ur‐3* and *Ur‐7* candidate genes represents an important advancement for plant pathology and breeding. Characterizing disease‐resistance (R) genes is challenging because they are typically located in structurally complex genomic regions, which contain high levels of sequence repetition and extensive, contiguous clusters of NB‐LRR genes. To overcome these challenges, we combined long‐read genome assemblies capable of resolving repetitive regions with recombination data from RIL populations for fine‐mapping, and integrated SNP‐ and *k*‐mer‐based GWAS to refine trait associations. For instance, combining *k*‐mer analysis with long‐read assemblies in the Andean diversity panel enabled the discovery of multiple anthracnose‐resistance genes that were not captured in existing reference genomes (Wiersma et al., 2024). Similarly, integrating long‐ and short‐read assemblies facilitated the identification of a specific TNL underlying the *I* gene associated with BCMV resistance (Alvarez‐Diaz et al., 2026).

Additionally, identifying candidate genes for both *Ur‐3* and *Ur‐7* generates an opportunity to develop more precise gene‐based markers and support MAS in common bean breeding programs for rust resistance. Although Ur3_Pv11_GeneR (50.2 Mb) and Ur7_Pv11_GeneR (32.8 Mb) markers are situated on the same chromosome, they are separated by a substantial physical distance (∼17.4 Mb; ∼32 cM based in O12 RIL population), indicative of weak genetic linkage and a relatively high recombination frequency between the loci. This is particularly valuable because greenhouse phenotyping is labor‐intensive, slow (typically requiring approximately 21 days), and constrained by the biotrophic nature of the rust pathogen, which is difficult to maintain and handle. As a result, most dry and snap bean breeders cannot routinely perform reliable rust phenotyping, making gene‐based markers a critical tool for advancing resistance breeding.

Although the genomic evidence provides strong support for these candidate genes, functional validation and broader genotype screening remain essential to confirm their identities and characterize allelic diversity. As additional reference genomes and pangenomes that retain these structurally variable loci become available, gene‐expression analyses will become more reliable, particularly for candidates embedded within regions affected by copy‐number or presence—absence variation. Previous transcriptomic studies related to rust resistance have largely relied on the Andean reference genome and have focused primarily on the *Ur‐3* and *Crg* (complements resistance gene for *Ur‐3*) loci, underscoring the need for expression analyses anchored to genomic assemblies that actually contain the full complement of resistance‐gene haplotypes of interest (Makhumbila et al., 2025; Todd et al., 2017).

Overall, our findings advance the genetic understanding of two major, *Ur‐3* and *Ur‐7*, rust‐resistance loci against *U. appendiculatus* and provide practical resources to support gene pyramiding and the development of more durable rust‐resistant bean varieties. Consequently, these resistance loci can be effectively pyramided through MAS with minimal constraint from linkage. Furthermore, the pipeline and genomic resources developed and compiled in this study provide a robust framework for the identification of other rust‐resistant genes, particularly those located in complex regions that are often underrepresented or absent in current reference genomes.

## AUTHOR CONTRIBUTIONS

**Alvaro Soler‐Garzón**: Conceptualization; data curation; formal analysis; investigation; methodology; validation; writing—original draft. **Giseli Valentini**: Investigation; methodology; writing—review and editing. **Marcial Pastor‐Corrales**: Investigation; methodology; writing—review and editing. **Ruifeng He**: Investigation; methodology; writing—review and editing. **Phillip N. Miklas**: Investigation; project administration; resources; writing—review and editing.

## CONFLICT OF INTEREST STATEMENT

The authors declare no conflicts of interest.

## Supporting information



Supplementary pdf file presenting Figures S1–S5, including statistical results from *k*‐mer and SNP genotyping analyses.

Supplementary xlsx file presenting Table S1‐S3, including SRA datasets, phenotypic data, and survey markers.

## Data Availability

The USDA Rattler sample is cataloged in NCBI under BioSample SAMN54680171 with SRA accession SRS28552282. Its Whole Genome Shotgun project has been deposited in DDBJ/ENA/GenBank under accession JBTRSO000000000, and the version described in this study is JBTRSO010000000.

## References

[tpg270253-bib-0001] Alonge, M. , Lebeigle, L. , Kirsche, M. , Jenike, K. , Ou, S. , Aganezov, S. , Wang, X. , Lippman, Z. B. , Schatz, M. C. , & Soyk, S. (2022). Automated assembly scaffolding using RagTag elevates a new tomato system for high‐throughput genome editing. Genome Biology, 23, 258. 10.1186/s13059-022-02823-7 36522651 PMC9753292

[tpg270253-bib-0071] Alvarez‐Diaz, J. C. , Soler‐Garzón, A. , Teano, G. , Verdenaud, M. , Roudaut, A. , Klopp, C. , Prejean, M.‐V. , Pflieger, S. , Porch, T. G. , Desbiez, C. , Latrasse, D. , Benhamed, M. , Delannoy, E. , Pedrosa‐Harand, A. , Gratias, A. , Miklas, P. N. , & Geffroy, V. (2026). The I gene defines a dynamic NLR cluster conferring broad potyvirus resistance in common bean. Nature Communications. In press. 10.1038/s41467-026-73550-x PMC13392006 42218130

[tpg270253-bib-0003] Andrews, S. (2010). FastQC: A quality control tool for high throughput sequence data . QUBES. https://qubeshub.org/resources/fastqc

[tpg270253-bib-0004] Bish, M. D. , Ramachandran, S. R. , Wright, A. , Lincoln, L. M. , Whitham, S. A. , Graham, M. A. , & Pedley, K. F. (2024). The soybean *Rpp3* gene encodes a TIR‐NBS‐LRR protein that confers resistance to *Phakopsora pachyrhizi* . Molecular Plant–Microbe Interactions, 37, 561–570. 38569009 10.1094/MPMI-01-24-0007-R

[tpg270253-bib-0005] Chen, S. , Zhou, Y. , Chen, Y. , & Gu, J. (2018). fastp: An ultra‐fast all‐in‐one FASTQ preprocessor. Bioinformatics, 34, i884–i890. 30423086 10.1093/bioinformatics/bty560 PMC6129281

[tpg270253-bib-0006] Cheng, H. , Asri, M. , Lucas, J. , Koren, S. , & Li, H. (2024). Scalable telomere‐to‐telomere assembly for diploid and polyploid genomes with a double graph. Nature Methods, 21, 967–970. 10.1038/s41592-024-02269-8 38730258 PMC11214949

[tpg270253-bib-0007] Cichy, K. A. , Caldas, G. V. , Snapp, S. S. , & Blair, M. W. (2009). QTL analysis of seed iron, zinc, and phosphorus levels in an Andean bean population. Crop Science, 49, 1742–1750. 10.2135/cropsci2008.10.0605

[tpg270253-bib-0008] Corut, A. K. , & Wallace, J. G. (2024). kGWASflow: A modular, flexible, and reproducible Snakemake workflow for k‐mers‐based GWAS. G3: Genes, Genomes, Genetics, 14, jkad246. 10.1093/g3journal/jkad246 PMC10755180 37976215

[tpg270253-bib-0009] De Coster, W. , D'Hert, S. , Schultz, D. T. , Cruts, M. , & Van Broeckhoven, C. (2018). NanoPack: Visualizing and processing long‐read sequencing data. Bioinformatics, 34, 2666–2669. 10.1093/BIOINFORMATICS/BTY149 29547981 PMC6061794

[tpg270253-bib-0010] Dixon, P. (2003). VEGAN, a package of R functions for community ecology. Journal of Vegetation Science, 14, 927–930. 10.1111/j.1654-1103.2003.tb02228.x

[tpg270253-bib-0011] Eitas, T. K. , & Dangl, J. L. (2010). NB‐LRR proteins: Pairs, pieces, perception, partners, and pathways. Current Opinion in Plant Biology, 13, 472–477. 10.1016/j.pbi.2010.04.007 20483655 PMC2910844

[tpg270253-bib-0012] Gabriel, L. , Brůna, T. , Hoff, K. J. , Ebel, M. , Lomsadze, A. , Borodovsky, M. , & Stanke, M. (2023). BRAKER3: Fully automated genome annotation using RNA‐seq and protein evidence with GeneMark‐ETP, AUGUSTUS, and TSEBRA. Genome Research, 34(5), 769–777. 10.1101/2023.06.10.544449 PMC11216308 38866550

[tpg270253-bib-0013] Gao, D. , Abernathy, B. , Rohksar, D. , Schmutz, J. , & Jackson, S. A. (2014). Annotation and sequence diversity of transposable elements in common bean (*Phaseolus vulgaris*). Frontiers in Plant Science, 5, 339. 10.3389/fpls.2014.00339 25071814 PMC4093653

[tpg270253-bib-0014] Grafton, K. F. , Weiser, G. C. , Littlefield, L. J. , & Stavely, J. R. (1985). Inheritance of resistance to two races of leaf rust in dry edible bean. Crop Science, 25, 537–539. 10.2135/cropsci1985.0011183x002500030025x

[tpg270253-bib-0015] Guy, L. , Roat Kultima, J. , & Andersson, S. G. E. (2010). genoPlotR: Comparative gene and genome visualization in R. Bioinformatics, 26, 2334–2335. 10.1093/bioinformatics/btq413 20624783 PMC2935412

[tpg270253-bib-0016] Hurtado‐Gonzales, O. P. , Valentini, G. , Gilio, T. A. S. , Martins, A. M. , Song, Q. , & Pastor‐Corrales, M. A. (2017). Fine mapping of *Ur*‐3, a historically important rust resistance locus in common bean. G3 Genes|Genomes|Genetics, 7, 557–569. 10.1534/g3.116.036061 28031244 PMC5295601

[tpg270253-bib-0017] Jones, P. , Binns, D. , Chang, H.‐Y , Fraser, M. , Li, W. , Mcanulla, C. , Mcwilliam, H. , Maslen, J. , Mitchell, A. , Nuka, G. , Pesseat, S. , Quinn, A. F. , Sangrador‐Vegas, A. , Scheremetjew, M. , Yong, S.‐Y. , Lopez, R. , & Hunter, S. (2014). InterProScan 5: Genome‐scale protein function classification. Bioinformatics, 30, 1236–1240. 10.1093/bioinformatics/btu031 24451626 PMC3998142

[tpg270253-bib-0018] Jung, G. , Coyne, D. P. , Bokosi, J. , Steadman, J. R. , & Nienhuis, J. (1998). Mapping genes for specific and adult plant resistance to rust and abaxial leaf pubescence and their genetic relationships using randomly amplified polymorphic DNA (RAPD) markers in common bean. Journal of the American Society for Horticultural Science, 123, 859–863.

[tpg270253-bib-0019] Jurado, M. , Die, J. V. , Ferreira, J. J. , & Campa, A. (2025). Chromosome‐level dataset from de novo assembly of a Fabada common bean genotype using Illumina and PacBio technologies. Data in Brief, 63, 112219. 10.1016/j.dib.2025.112219 41334509 PMC12666108

[tpg270253-bib-0020] Kelly, J. D. , Stavely, J. R. , & Miklas, P. N. (1996). Proposed symbols for rust resistance genes. Annual Report of the Bean Improvement Cooperative, 39, 25–31.

[tpg270253-bib-0021] Kim, D. , Paggi, J. M. , Park, C. , Bennett, C. , & Salzberg, S. L. (2019). Graph‐based genome alignment and genotyping with HISAT2 and HISAT‐genotype. Nature Biotechnology, 37, 907–915. 10.1038/s41587-019-0201-4 PMC7605509 31375807

[tpg270253-bib-0022] Kokot, M. , Długosz, M. , & Deorowicz, S. (2017). KMC 3: Counting and manipulating *k*‐mer statistics. Bioinformatics, 33, 2759–2761. 10.1093/bioinformatics/btx304 28472236

[tpg270253-bib-0023] Langmead, B. , & Salzberg, S. L. (2012). Fast gapped‐read alignment with Bowtie 2. Nature Methods, 9, 357–359. 10.1038/nmeth.1923 22388286 PMC3322381

[tpg270253-bib-0024] Li, H. , Handsaker, B. , Wysoker, A. , Fennell, T. , Ruan, J. , Homer, N. , Marth, G. , Abecasis, G. , & Durbin, R. (2009). The sequence alignment/map format and SAMtools. Bioinformatics, 25, 2078–2079. 10.1093/bioinformatics/btp352 19505943 PMC2723002

[tpg270253-bib-0025] Liao, X. , Hu, K. , Salhi, A. , Zou, Y. , Wang, J. , & Gao, X. (2022). MsRepDB: A comprehensive repetitive sequence database of over 80000 species. Nucleic Acids Research, 50, D236–D245. 10.1093/nar/gkab1089 34850956 PMC8728181

[tpg270253-bib-0026] Liebenberg, M. M. , & Pretorius, Z. A. (2004). Inheritance of resistance to *Uromyces appendiculatus* in the South African dry bean cultivar Kranskop. South African Journal of Plant and Soil, 21, 245–250. 10.1080/02571862.2004.10635057

[tpg270253-bib-0027] Liebenberg, M. M. , & Pretorius, Z. A. (2010). Common bean rust: Pathology and control. Horticultural Reviews, 37, 1–99.

[tpg270253-bib-0028] Lipka, A. E. , Tian, F. , Wang, Q. , Peiffer, J. , Li, M. , Bradbury, P. J. , Gore, M. A. , Buckler, E. S. , & Zhang, Z. (2012). GAPIT: Genome association and prediction integrated tool. Bioinformatics, 28(18), 2397–2399. 10.1093/bioinformatics/bts444 22796960

[tpg270253-bib-0029] Lobaton, J. D. , Miller, T. , Gil, J. , Ariza, D. , De La Hoz, J. F. , Soler, A. , Beebe, S. , Duitama, J. , Gepts, P. , & Raatz, B. (2018). Resequencing of common bean identifies regions of inter–genepool introgression and provides comprehensive resources for molecular breeding. The Plant Genome, 11, 170068. 10.3835/plantgenome2017.08.0068 PMC12810085 30025029

[tpg270253-bib-0030] Luannfinke, M. , Coyne, D. P. , & Steadman, J. R. (1986). The inheritance and association of resistance to rust, common bacterial blight, plant habit, and foliar abnormalities in *Phaseolus vulgaris* L. Euphytica, 35, 969–982. 10.1007/BF00028607

[tpg270253-bib-0031] Makhumbila, P. , Rauwane, M. , Muedi, H. , & Figlan, S. (2025). Insights of *Phaseolus vulgaris*’ response to infection by *Uromyces appendiculatus* using an RNA‐seq approach. Frontiers in Plant Science, 16, 1557954. 10.3389/fpls.2025.1557954 40530296 PMC12171439

[tpg270253-bib-0032] Manni, M. , Berkeley, M. R. , Seppey, M. , Simão, F. A. , & Zdobnov, E. M. (2021). BUSCO update: Novel and streamlined workflows along with broader and deeper phylogenetic coverage for scoring of eukaryotic, prokaryotic, and viral genomes. Molecular Biology and Evolution, 38, 4647–4654. 10.1093/molbev/msab199 34320186 PMC8476166

[tpg270253-bib-0033] Martin, M. (2011). Cutadapt removes adapter sequences from high‐throughput sequencing reads. EMBnet.journal, 17, 10.

[tpg270253-bib-0034] Mcclean, P. E. , Lee, R. , Howe, K. , Osborne, C. , Grimwood, J. , Levy, S. , Haugrud, A. P. , Plott, C. , Robinson, M. , Skiba, R. M. , Tanha, T. , Zamani, M. , Thannhauser, T. W. , Glahn, R. P. , Schmutz, J. , Osorno, J. M. , & Miklas, P. N. (2022). The common bean V gene encodes flavonoid 3′5′ hydroxylase: A major mutational target for flavonoid diversity in angiosperms. Frontiers in Plant Science, 13, 869582. 10.3389/fpls.2022.869582 35432409 PMC9009181

[tpg270253-bib-0035] Meyers, B. C. , Dickerman, A. W. , Michelmore, R. W. , Sivaramakrishnan, S. , Sobral, B. W. , & Young, N. D. (1999). Plant disease resistance genes encode members of an ancient and diverse protein family within the nucleotide‐binding superfamily. The Plant Journal, 20, 317–332. 10.1046/j.1365-313x.1999.00606.x 10571892

[tpg270253-bib-0036] Miklas, P. N. , Delorme, R. , Stone, V. , Daly, M. J. , Stavely, J. R. , Steadman, J. R. , Bassett, M. J. , & Beaver, J. S. (2000). Bacterial, fungal, and viral disease resistance loci mapped in a recombinant inbred common bean population ('Dorado’/XAN 176). Journal of the American Society for Horticultural Science, 125, 476–481. 10.21273/jashs.125.4.476

[tpg270253-bib-0037] Miklas, P. N. , Pastor‐Corrales, M. A. , Jung, G. , Coyne, D. P. , Kelly, J. D. , McClean, P. E. , & Gepts, P. (2002). Comprehensive linkage map of bean rust resistance genes. Annual Report—Bean Improvement Cooperative, 45, 125–129.

[tpg270253-bib-0038] Miklas, P. N. , Soler‐Garzón, A. , Valentini, G. , & Pastor‐Corrales, M. (2023). Registration of ‘USDA Rattler’ pinto bean. Journal of Plant Registrations, 17, 1–9. 10.1002/plr2.20289

[tpg270253-bib-0039] Nadeem, M. A. , Yeken, M. Z. , Shahid, M. Q. , Habyarimana, E. , Yılmaz, H. , Alsaleh, A. , Hatipoğlu, R. , Çilesiz, Y. , Khawar, K. M. , Ludidi, N. , Ercişli, S. , Aasim, M. , Karaköy, T. , & Baloch, F. S. (2021). Common bean as a potential crop for future food security: An overview of past, current and future contributions in genomics, transcriptomics, transgenics and proteomics. Biotechnology & Biotechnological Equipment, 35, 759–787. 10.1080/13102818.2021.1920462

[tpg270253-bib-0040] Park, S. O. , Coyne, D. P. , Steadman, J. R. , & Skroch, P. W. (2003). Mapping of the *Ur‐7* gene for specific resistance to rust in common bean. Crop Science, 43, 1470–1476. 10.2135/cropsci2003.1470

[tpg270253-bib-0041] Park, S. O. , Steadman, J. R. , Coyne, D. P. , & Crosby, K. M. (2008). Development of a coupling‐phase SCAR marker linked to the *Ur‐7* rust resistance gene and its occurrence in diverse common bean lines. Crop Science, 48, 357–363. 10.2135/cropsci2007.03.0179

[tpg270253-bib-0069] Pastor‐Corrales, M. A. (2005). Inheritance of resistance in PI 260418 an Andean bean resistant to most races of the bean rust pathogen. Annual Report of the Bean Improvement Cooperative, 48, 134–135.

[tpg270253-bib-0042] Pastor‐Corrales, M. A. , Rayapati, J. , Osorno, J. M. , Kelly, J. D. , Wright, E. M. , Brick, M. A. , Markell, S. G. , & Goswami, R. S. (2010). Reaction of common bean cultivars to two new races of rust pathogen from Michigan and North Dakota. Annual Report of the Bean Improvement Cooperative, 53, 66–67.

[tpg270253-bib-0070] Perry, G. , DiNatale, C. , Xie, W. , Navabi, A. , Reinprecht, Y. , Crosby, W. , Yu, K. , Shi, C. , & Pauls, K. P. (2013). A comparison of the molecular organization of genomic regions associated with resistance to common bacterial blight in two Phaseolus vulgaris genotypes. Frontiers in Plant Science, 4, 00318. 10.3389/fpls.2013.00318 PMC3756299 24009615

[tpg270253-bib-0043] Roy, J. , Sreedasyam, A. , Osborne, C. , Lee, R. , & McClean, P. E. (2025). Seed coat transcriptomic profiling of 5–593, a genotype important for genetic studies of seed coat color and patterning in common bean (*Phaseolus vulgaris* L.). BMC Plant Biology, 25(1), 284. 10.1186/s12870-025-06282-7 40038560 PMC11881399

[tpg270253-bib-0044] Schmutz, J. , Mcclean, P. E. , Mamidi, S. , Wu, G. A. , Cannon, S. B. , Grimwood, J. , Jenkins, J. , Shu, S. , Song, Q. , Chavarro, C. , Torres‐Torres, M. , Geffroy, V. , Moghaddam, S. M. , Gao, D. , Abernathy, B. , Barry, K. , Blair, M. , Brick, M. A. , Chovatia, M. , … Jackson, S. A. (2014). Reference genome for common bean and genome‐wide analysis of dual domestications. Nature Genetics, 46, 707–713. 10.1038/ng.3008 24908249 PMC7048698

[tpg270253-bib-0045] Schwartz, H. F. , & Pastor‐ Corrales, M. A. (1989). Bean production problems in the tropics. CIAT.

[tpg270253-bib-0046] Singh, S. P. , Gepts, P. , & Debouck, D. G. (1991). Races of common bean (*Phaseolus vulgaris*, Fabaceae). Economic Botany, 45, 379–396.

[tpg270253-bib-0047] Soler‐Garzón, A. , McClean, P. E. , & Miklas, P. N. (2021a). Genome‐wide association mapping of *bc‐1* and *bc‐u* reveals candidate genes and new adjustments to the host‐pathogen interaction for resistance to bean common mosaic necrosis virus in common bean. Frontiers in Plant Science, 12, 769247. 10.3389/fpls.2021.699569 34267774 PMC8277298

[tpg270253-bib-0048] Soler‐Garzón, A. , McClean, P. E. , & Miklas, P. N. (2021b). Coding mutations in vacuolar protein‐sorting 4 AAA+ ATPase endosomal sorting complexes required for transport protein homologs underlie *bc‐2* and new *bc‐4* gene conferring resistance to bean common mosaic virus in common bean. Frontiers in Plant Science, 12, 769247. 10.3389/fpls.2021.769247 34966401 PMC8710759

[tpg270253-bib-0049] Song, Q. , Jia, G. , Hyten, D. L. , Jenkins, J. , Hwang, E.‐Y. , Schroeder, S. G. , Osorno, J. M. , Schmutz, J. , Jackson, S. A. , Mcclean, P. E. , & Cregan, P. B. (2015). SNP assay development for linkage map construction, anchoring whole genome sequence and other genetic and genomic applications in common bean. G3 Genes|Genomes|Genetics, 5, 2285–2290. 10.1534/g3.115.020594 26318155 PMC4632048

[tpg270253-bib-0050] Stavely, J. R. (1984). Pathogenic specialization in *Uromyces phaseoli* in the United States and rust resistance in beans. Plant Disease, 68(1), 95. 10.1094/PD-68-95

[tpg270253-bib-0051] Stavely, J. R. (1990). Genetics of rust resistance in *Phaseolus vulgaris* plant introduction ‘PI 181996’. Phytopathology, 80, 1056.

[tpg270253-bib-0052] Stavely, J. R. , & Pastor‐Corrales, M. A. (1989). Rust. In H. F. Schwartz & M. A. Pastor‐Corrales (Eds.), Bean production problems in the tropics (2nd ed., pp. 159–194). Centro Internacional de Agricultura Tropical (CIAT).

[tpg270253-bib-0053] Tello, D. , Gonzalez‐Garcia, L. N. , Gomez, J. , Zuluaga‐Monares, J. C. , Garcia, R. , Angel, R. , Mahecha, D. , Duarte, E. , Leon, M. D. R. , Reyes, F. , Escobar‐Velásquez, C. , Linares‐Vásquez, M. , Cardozo, N. , & Duitama, J. (2023). NGSEP 4: Efficient and accurate identification of orthogroups and whole‐genome alignment. Molecular Ecology Resources, 23(3), 712–724. 36377253 10.1111/1755-0998.13737

[tpg270253-bib-0054] Todd, A. , Donofrio, N. , Sripathi, V. , Mcclean, P. , Lee, R. , Pastor‐Corrales, M. , & Kalavacharla, V. (2017). Marker‐assisted molecular profiling, deletion mutant analysis, and RNA‐seq reveal a disease resistance cluster associated with *Uromyces appendiculatus* infection in common bean (*Phaseolus vulgaris* L.). International Journal of Molecular Sciences, 18, 1109. 10.3390/ijms18061109 28545258 PMC5485933

[tpg270253-bib-0055] Trapp, J. , Urrea, C. , Cregan, P. , & Miklas, P. N. (2015). Quantitative trait loci for yield under multiple stress and drought conditions in a dry bean population. Crop Science, 55, 1596–1607. 10.2135/cropsci2014.11.0792

[tpg270253-bib-0056] Untergasser, A. , Cutcutache, I. , Koressaar, T. , Ye, J. , Faircloth, B. C. , Remm, M. , & Rozen, S. G. (2012). Primer3—New capabilities and interfaces. Nucleic Acids Research, 40, e115. 10.1093/nar/gks596 22730293 PMC3424584

[tpg270253-bib-0057] Valentini, G. , Hurtado‐Gonzales, O. P. , Xavier, L. F. S. , He, R. , Gill, U. , Song, Q. , & Pastor‐Corrales, M. (2025a). Fine mapping of the unique *Ur‐11* gene conferring broad resistance to the rust pathogen of common bean. Theoretical and Applied Genetics, 138, 64. 10.1007/s00122-025-04856-5 40035870

[tpg270253-bib-0058] Valentini, G. , Pastor‐Corrales, M. A. , Hurtado‐Gonzales, O. P. , Tamang, P. , & Song, Q. (2025b). Unraveling the epistatic interactions of major rust resistance genes in common bean. European Journal of Plant Pathology, 173, 749–759.

[tpg270253-bib-0059] Valentini, G. , Pastor‐Corrales, M. A. , Hurtado‐Gonzales, O. P. , Xavier, L. F. S. , Gill, U. , & Song, Q. (2025c). Characterization and mapping of a rust resistance locus in the common bean landrace G19833. G3: Genes, Genomes, Genetics, 15, jkaf168. 10.1093/g3journal/jkaf168 40690558 PMC12405894

[tpg270253-bib-0060] Van Ooijen, G. , Mayr, G. , Kasiem, M. M. A. , Albrecht, M. , Cornelissen, B. J. C. , & Takken, F. L. W. (2008). Structure‐function analysis of the NB‐ARC domain of plant disease resistance proteins. Journal of Experimental Botany, 59, 1383–1397. 10.1093/jxb/ern045 18390848

[tpg270253-bib-0061] Vasconcellos, R. C. , Oraguzie, O. B. , Soler, A. , Arkwazee, H. , Myers, J. R. , Ferreira, J. J. , Song, Q. , McClean, P. , & Miklas, P. N. (2017). Meta‐QTL for resistance to white mold in common bean. PLoS ONE, 12(2), e0171685. 10.1371/journal.pone.0171685 28199342 PMC5310892

[tpg270253-bib-0062] Viscarra‐Torrico, R. C. , Pajak, A. , Garzón, A. S. , Zhang, B. , Pandurangan, S. , Diapari, M. , Song, Q. , Conner, R. L. , House, J. D. , Miklas, P. N. , Hou, A. , & Marsolais, F. (2021). Common bean (*Phaseolus vulgaris* L.) with increased cysteine and methionine concentration. Legume Science, 3, 1–12. 10.1002/leg3.103

[tpg270253-bib-0063] Vlasova, A. , Capella‐Gutiérrez, S. , Rendón‐Anaya, M. , Hernández‐Oñate, M. , Minoche, A. E. , Erb, I. , Câmara, F. , Prieto‐Barja, P. , Corvelo, A. , Sanseverino, W. , Westergaard, G. , Dohm, J. C. , Pappas, G. J. , Saburido‐Alvarez, S. , Kedra, D. , Gonzalez, I. , Cozzuto, L. , Gómez‐Garrido, J. , Aguilar‐Morón, M. A. , … Guigó, R. (2016). Genome and transcriptome analysis of the Mesoamerican common bean and the role of gene duplications in establishing tissue and temporal specialization of genes. Genome Biology, 17, 32. 10.1186/s13059-016-0883-6 26911872 PMC4766624

[tpg270253-bib-0064] Voichek, Y. , & Weigel, D. (2020). Identifying genetic variants underlying phenotypic variation in plants without complete genomes. Nature Genetics, 52(5), 534–540. 32284578 10.1038/s41588-020-0612-7 PMC7610390

[tpg270253-bib-0065] Wang, J. , Chuang, K. , Ahluwalia, M. , Patel, S. , Umblas, N. , Mirel, D. , Higuchi, R. , & Germer, S. (2005). High‐throughput SNP genotyping by single‐tube PCR with T_m_‐shift primers. Biotechniques, 39, 885–893. 10.2144/000112028 16382908

[tpg270253-bib-0066] Wang, Y. , Hao, X. , Chen, C. , Wang, H. , Gao, P. , Yang, X. , Dong, X. , Qin, H. , Li, M. , Hou, S. , Jian, J. , Chang, J. , Wu, J. , & Mu, Z. (2025). Telomere‐to‐telomere genome of common bean (*Phaseolus vulgaris* L., YP4). Gigascience, 14, giaf001. 10.1093/gigascience/giaf001 40366866 PMC12077395

[tpg270253-bib-0067] Wiersma, A. T. , Hamilton, J. P. , Vaillancourt, B. , Brose, J. , Awale, H. E. , Wright, E. M. , Kelly, J. D. , & Buell, C. R (2024). *k*‐mer genome‐wide association study for anthracnose and BCMV resistance in a *Phaseolus vulgaris* Andean Diversity Panel. The Plant Genome, 17(4), e20523. 10.1002/tpg2.20523 39397345 PMC11628888

[tpg270253-bib-0068] Yin, L. (2016). CMplot: Circular and rectangular Manhattan plot . GitHub. https://github.com/YinLiLin/CMplot?tab=readme‐ov‐file

