# Supplementary material for: Integrative genomic analyses reveal candidate genes for the Ur‐3 and Ur‐7 rust resistance loci in common bean
Source: Plant Genome. 2026 May 23;19(2):e70253. doi: 10.1002/tpg2.70253 (PMC13492622; doi:10.1002/tpg2.70253)
Supplement: Supplementary file 1 [file TPG2-19-e70253-s002.pdf]

Figure Supplementary 1

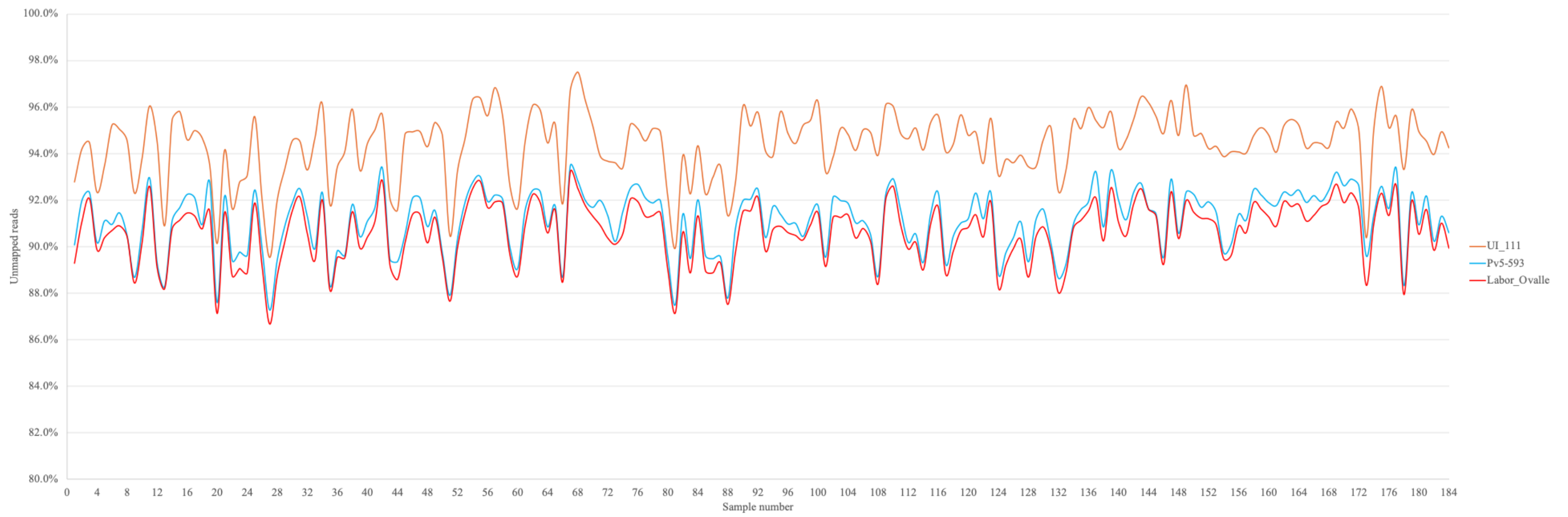

Figure S1. (A) Proportion of uniquely mapped and multi-mapped paired-end reads (y-axis) aggregated for each DDP accession (x-axis) across the Middle American reference genomes. Orange, blue, and red lines represent the percentage of short reads mapped per DDP accession to the UI111, Pv5-593, and Labor Ovalle reference genomes, respectively.

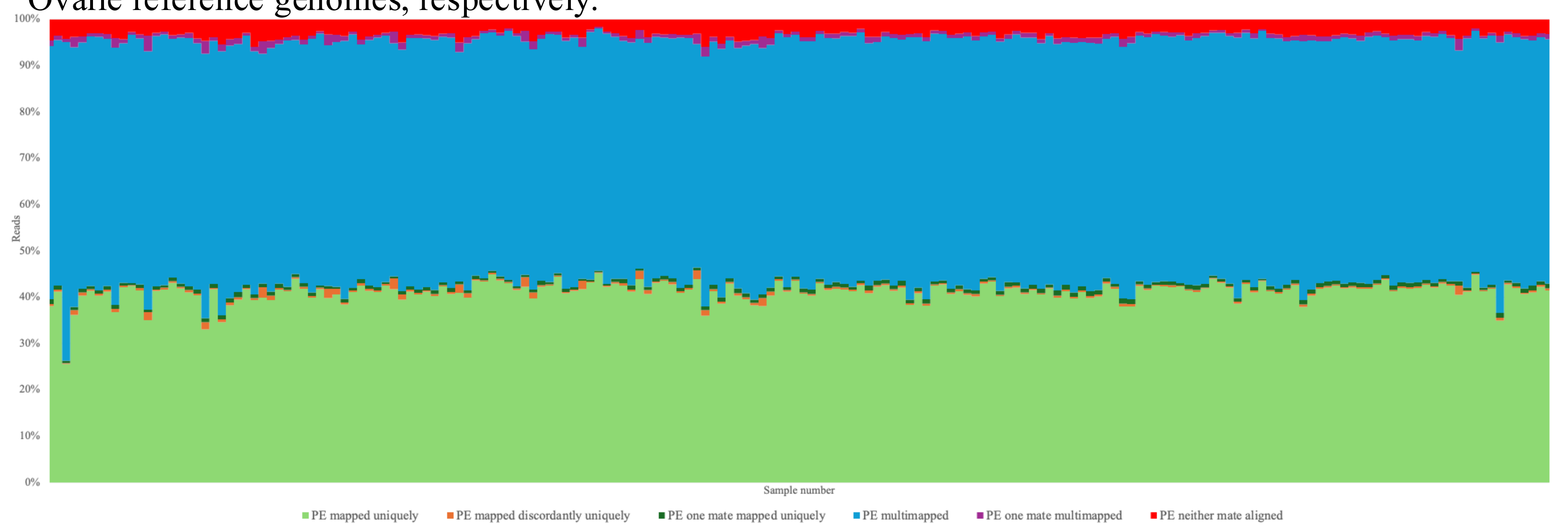

Figure S1. (B) Bowtie2 mapping summary for sequencing reads from DDP accessions aligned to the UI111 reference genome. Categories include uniquely mapped reads (light green), discordant unique reads (orange), single-mate mapped reads (dark green), multimapped reads (blue), single-mate multimapped reads (purple), and unmapped reads (red). The y-axis shows percentages, and each bar on the x-axis represents an individual sample.

Figure Supplementary 2

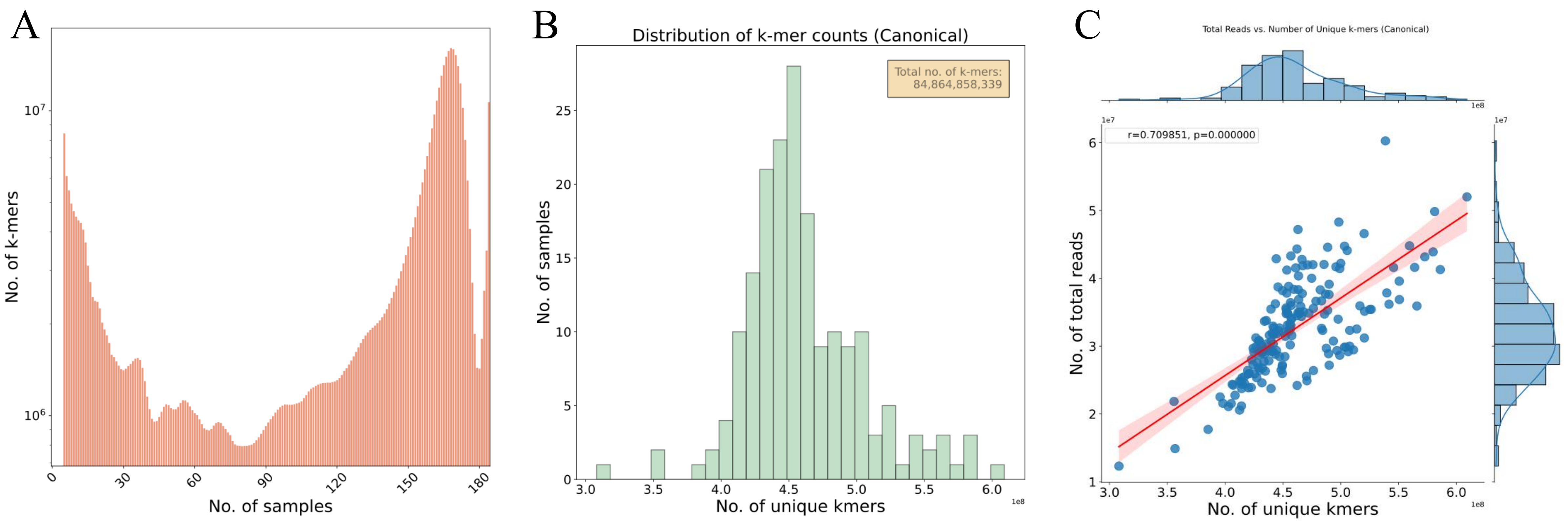

Figure S2. Outputs from the *k*-mer analysis of the DDP panel.
(A) Bar plot showing the number of *k*-mers occurring in exactly 184 DDP lines. Only *k*-mers retained after the initial filtering step are shown.
(B) Histogram displaying the distribution of canonical *k*-mer counts per sample, with the x-axis representing the number of unique *k*-mers and the y-axis the number of DDP lines. The total number of canonical unique *k*-mers is indicated in the legend.
(C) Joint plot illustrating the relationship between the number of canonical unique *k*-mers and total read counts per sample. The red line indicates the linear regression fit, with Pearson's *r* and *p*-value shown. Marginal distributions for both axes are displayed along the top and right margins.

Figure Supplementary 3

A

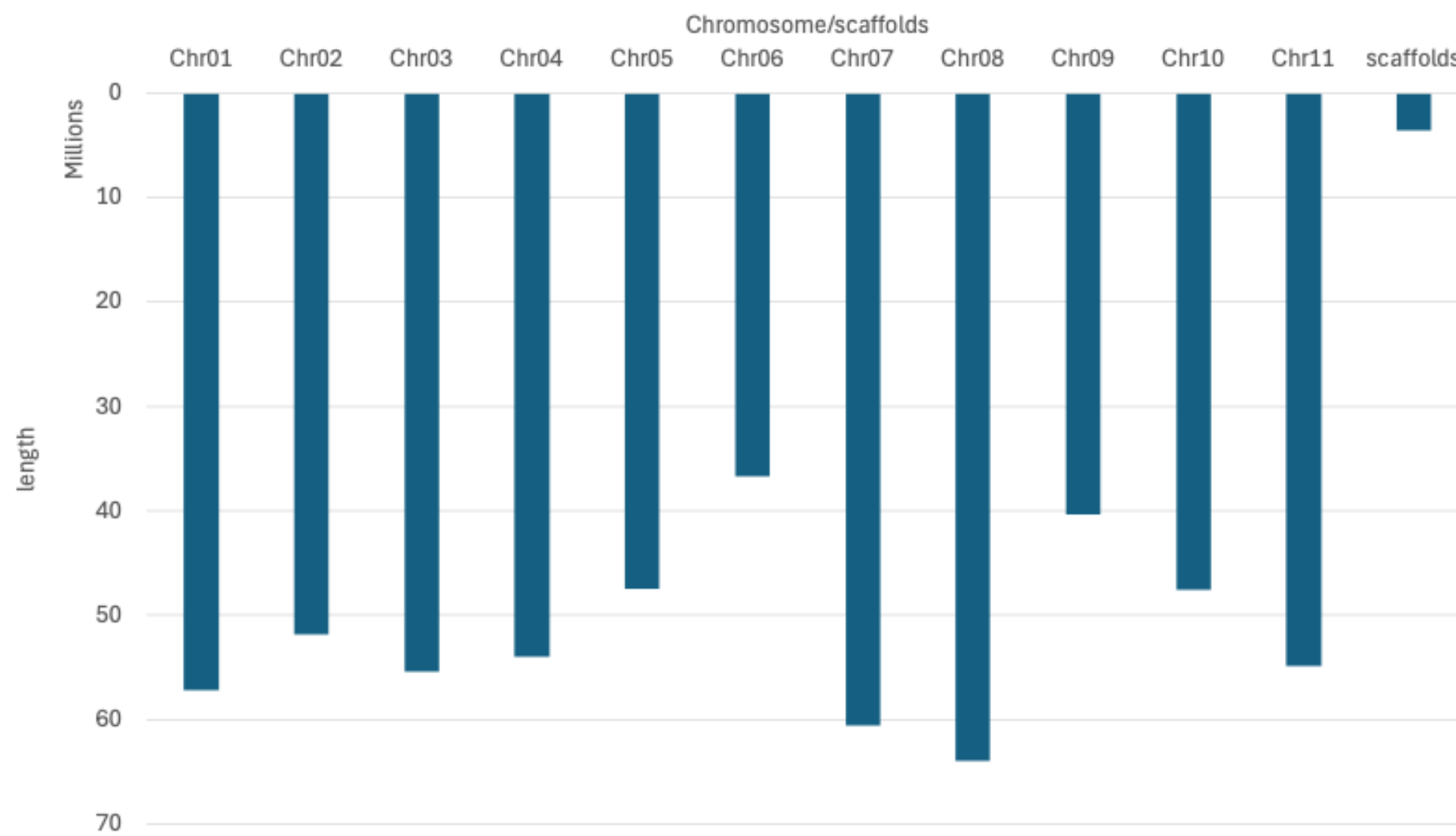

Figure 3A. Bar chart showing the lengths of chromosomes (Pv01–Pv11) in USDA Rattler assembly and unplaced scaffolds in megabases. Chromosome sizes range from ~35–60 Mb, and the total assembly length is 573.5 Mb.

B

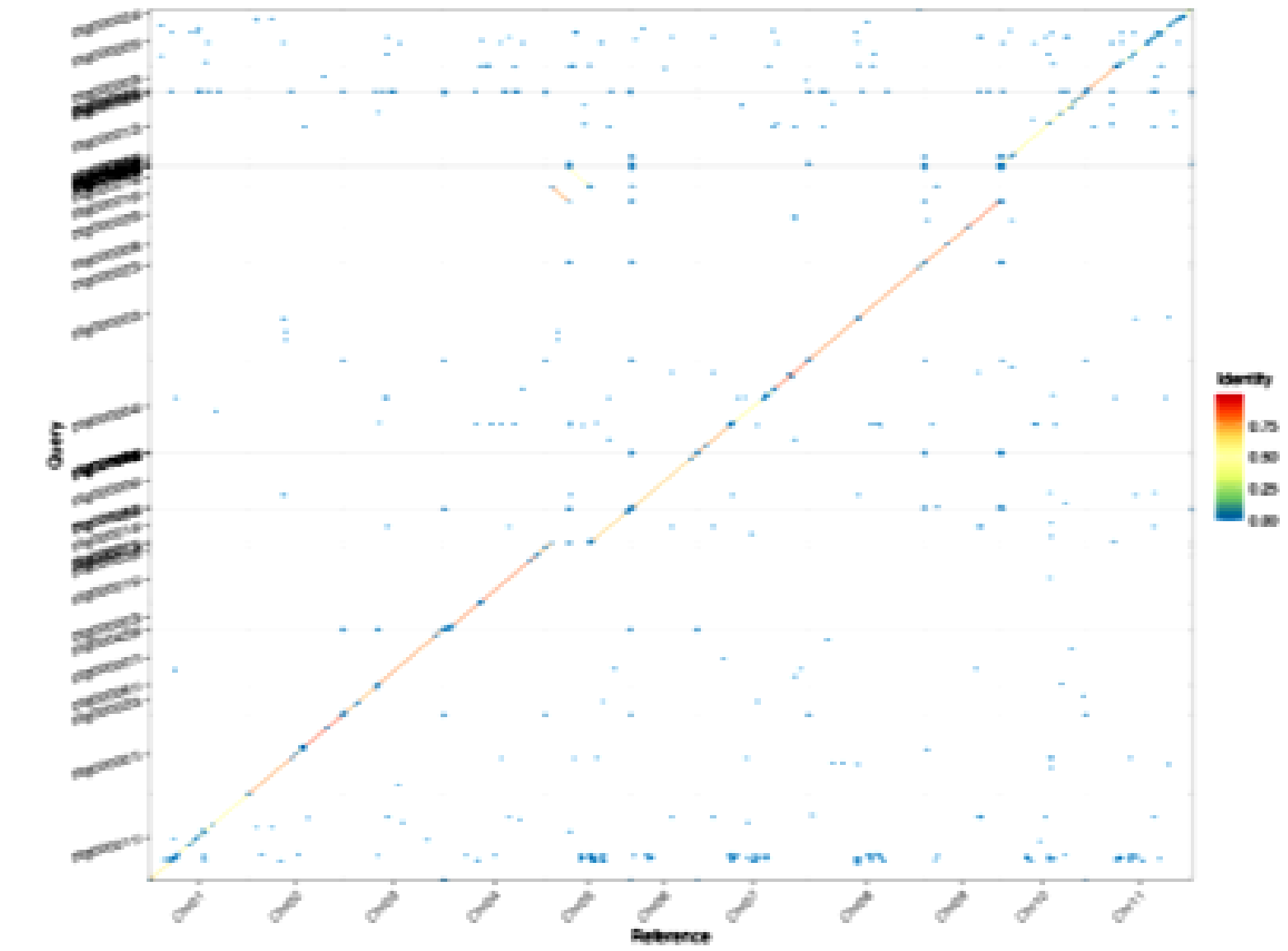

Figure 3B. Genome synteny dot plot showing pairwise alignments between Pv5-593 reference and query USDA Rattler contigs; diagonal signals indicate conserved collinearity, while scattered points reflect rearrangements or duplicated regions.

C

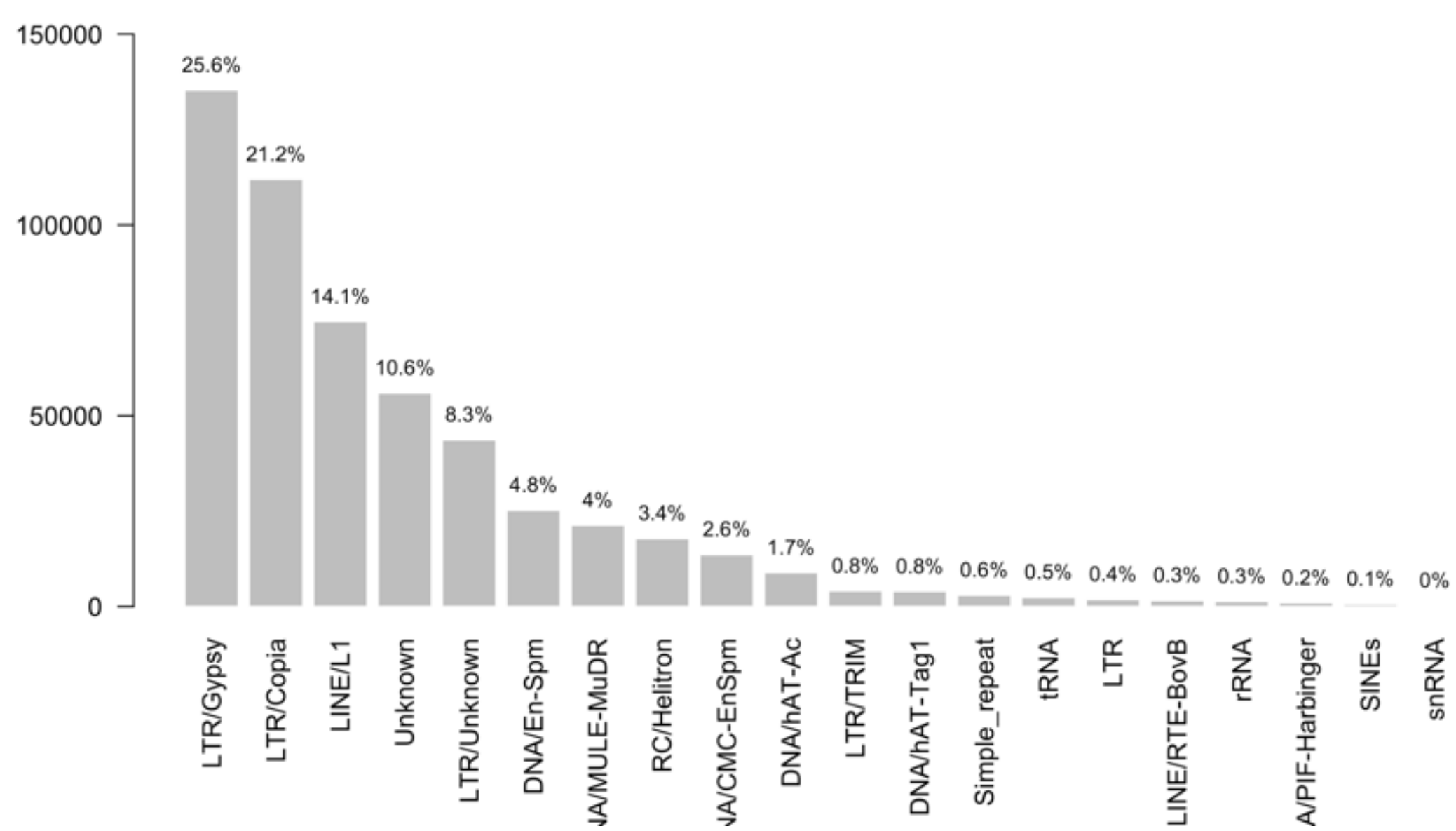

Figure 3C. Proportion of major transposable element (TE) families in the USDA Rattler genome, with LTR/Gypsy and LTR/Copia representing the largest fractions.

D

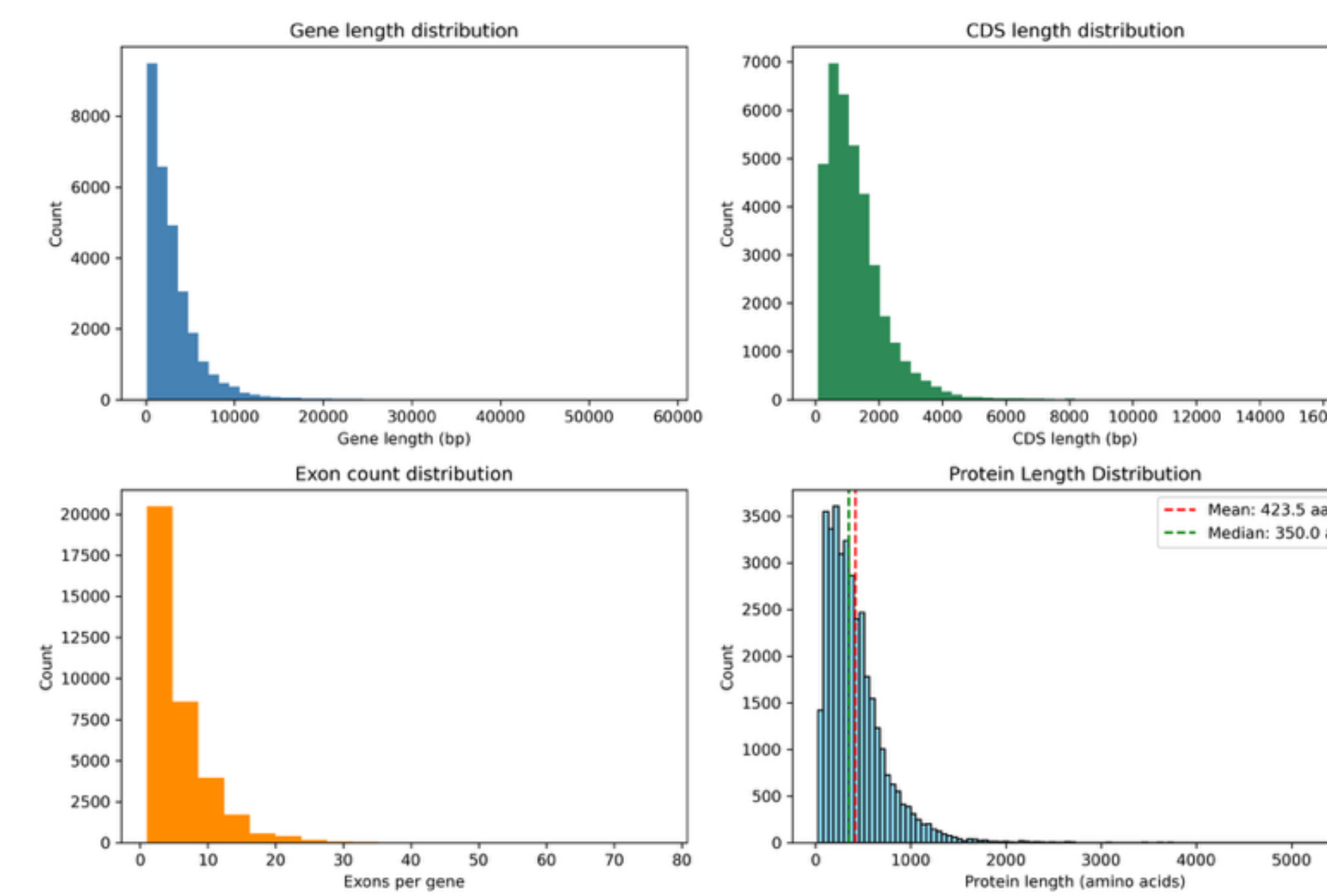

Figure 3D. Gene annotation in USDA Rattler genome. **Top left:** Gene length distribution. **Top right:** CDS length distribution. **Bottom left:** Exon count per gene. **Bottom right:** Protein length distribution with mean (red) and median (green) indicated.

Figure Supplementary 4

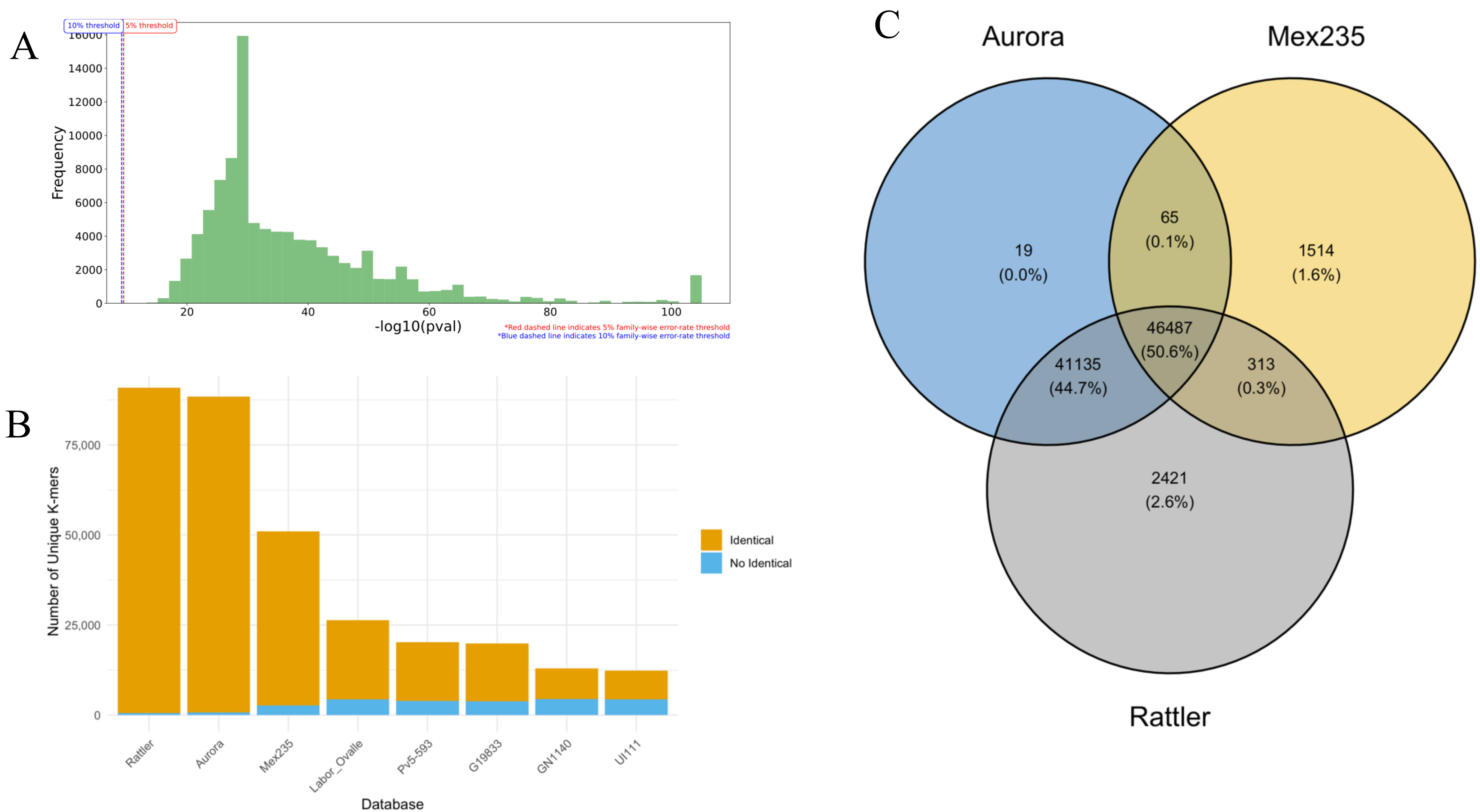

Figure S4. Significant *k*-mers associated to *Ur-3* gene. **(A)** Histogram showing the distribution of $-\log_{10}$ *P*-values for all detected *k*-mers associated with the *Ur-3* resistance gene. **(B)** BLAST results of significant *k*-mers aligned to reference genomes, showing the number of *k*-mers identical to each reference sequence. **(C)** Venn diagram showing *k*-mers shared among USDA Rattler, Aurora, and MEX 235 genotypes carrying the *Ur-3* resistance gene, highlighting common and unique sequence patterns.

Figure Supplementary 5

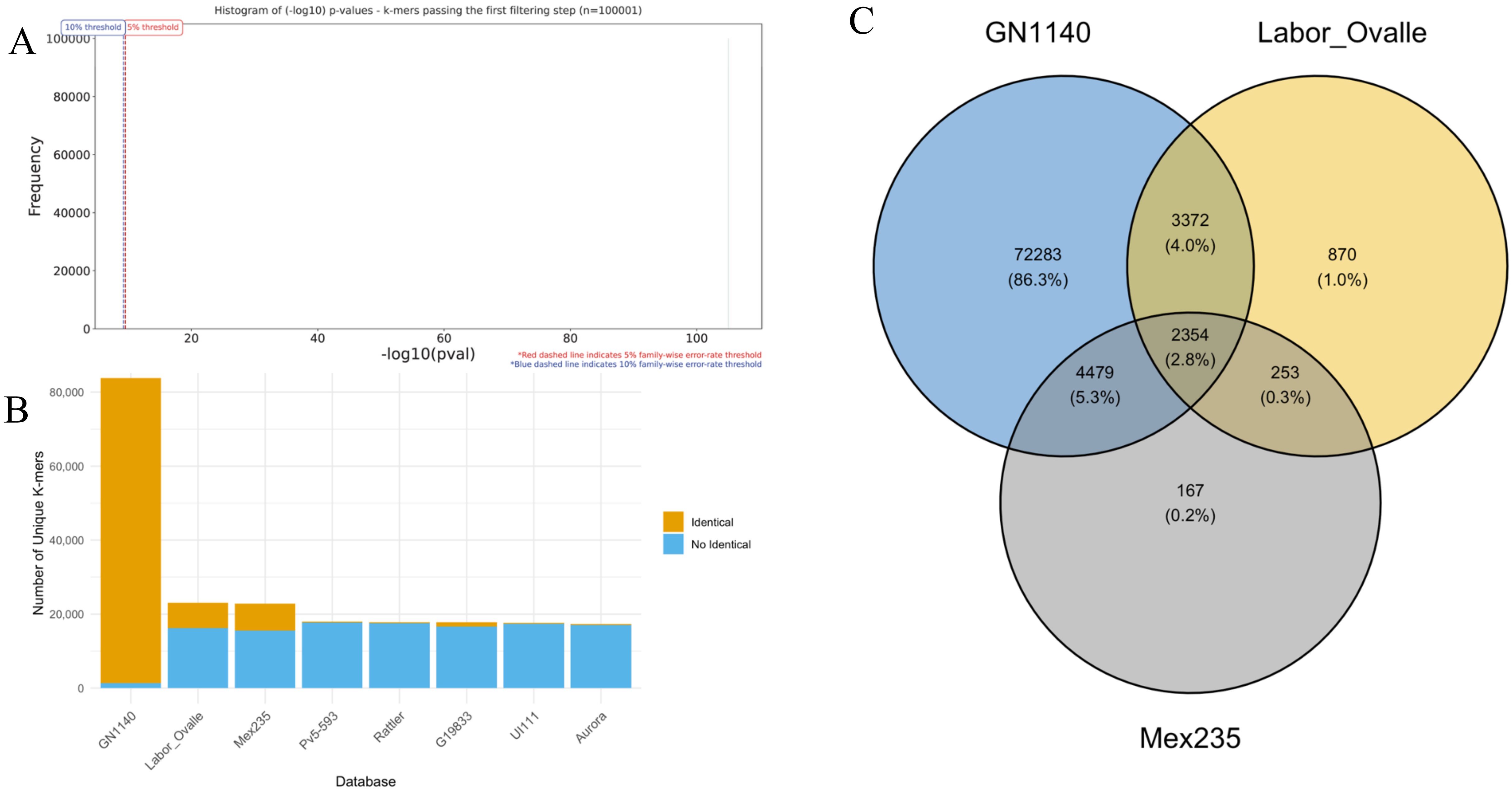

Figure S5. Significant *k*-mers associated to *Ur-7* gene. **(A)** Histogram showing the distribution of $-\log_{10}$ *P*-values for all detected *k*-mers associated with the *Ur-7* resistance gene. **(B)** BLAST results of significant *k*-mers aligned to reference genomes, showing the number of *k*-mers identical to each reference sequence. **(C)** Venn diagram showing unique *k*-mers in GN 1140 and discarding *k*-mers in Labor Ovalle (*ur-7*) and MEX 235 (*ur-7*).
